# Risk Factors and Incidence for In-Stent Restenosis with Drug-Eluting Stent: A Systematic Review and Meta-Analysis

**DOI:** 10.31083/j.rcm2512458

**Published:** 2024-12-24

**Authors:** Birong Liu, Meng Li, Jia Liu, Lihua Xie, Jiaqi Li, Yong Liu, Chaofeng Niu, Di Xiao, Jingen Li, Lijing Zhang

**Affiliations:** ^1^Department of Cardiovascular Medicine, Dongzhimen Hospital, Beijing University of Chinese Medicine, 100700 Beijing, China; ^2^Department of Cardiovascular Medicine, Xi’an Hospital of Traditional Chinese Medicine, 710001 Xi'an, Shaanxi, China; ^3^Department of Cardiovascular Medicine, Dongfang Hospital, Beijing University of Chinese Medicine, 100078 Beijing, China

**Keywords:** drug-eluting stent, in-stent restenosis, incidence, risk factors, meta-analysis

## Abstract

**Background:**

Despite significant reductions in in-stent restenosis (ISR) incidence with the adoption of drug-eluting stents (DES) over bare metal stents (BMS), ISR remains an unresolved issue in the DES era. The risk factors associated with DES-ISR have not been thoroughly analyzed. This meta-analysis aims to identify the key factors and quantify their impact on DES-ISR.

**Methods:**

We conducted comprehensive literature searches in PubMed, EMBASE, Cochrane, and Web of Science up to 28 February 2023, to identify studies reporting risk factors for DES-ISR. Meta-analysis was performed on risk factors reported in two or more studies to determine their overall effect sizes.

**Results:**

From 4357 articles screened, 17 studies were included in our analysis, evaluating twenty-four risk factors for DES-ISR through meta-analysis. The pooled incidence of DES-ISR was approximately 13%, and significant associations were found with seven risk factors. Ranked risk factors included diabetes mellitus (odds ratio [OR]: 1.46; 95% confidence interval [CI]: 1.14–1.87), stent length (OR: 1.026; 95% CI: 1.003–1.050), number of stents (OR: 1.62; 95% CI: 1.11–2.37), involvement of the left anterior descending artery (OR: 1.56; 95% CI: 1.25–1.94), lesion length (OR: 1.016; 95% CI: 1.008–1.024), medical history of myocardial infarction (OR: 1.79; 95% CI: 1.12–2.86) and previous percutaneous coronary intervention (OR: 1.97; 95% CI: 1.53–2.55). Conversely, a higher left ventricular ejection fraction was identified as a protective factor (OR: 0.985; 95% CI: 0.972–0.997).

**Conclusions:**

Despite advancements in stent technology, the incidence of ISR remains a significant clinical challenge. Our findings indicate that patient characteristics, lesion specifics, stent types, and procedural factors all contribute to DES-ISR development. Proactive strategies for early identification and management of these risk factors are essential to minimize the risk of ISR following DES interventions.

**The PROSPERO Registration:**

CRD42023427398, https://www.crd.york.ac.uk/PROSPERO/display_record.php?RecordID=427398.

## 1. Introduction

Since the introduction of percutaneous transluminal coronary angioplasty in 1977, interventional cardiology has evolved rapidly. Percutaneous coronary intervention (PCI) has adopted stents as a cornerstone of primary treatment for coronary artery disease (CAD) [1]. This progression has significantly enhanced the success of coronary revascularization. However, in-stent restenosis (ISR), defined as a diameter stenosis of ≥50% within the stented segment or within 5 mm proximal or distal to the stent, remains a persistent challenge in PCI [2, 3]. Over the last two decades, numerous technical advancements have been developed to mitigate ISR [4], involving the evolution of materials from simple balloons and bare metal stents (BMS) to sophisticated drug-eluting stents (DES), drug-coated balloons (DCB), and bioresorbable scaffolds (BRS) [5].

Despite the significant reduction in ISR with the advent of DES which release anti-inflammatory, immunomodulatory, or antiproliferative agents, 5–10% of patients receiving DES are still at risk of ISR [6, 7, 8]. Meanwhile, with the growing use of DES and the increasing number of complex lesions treated, the number of patients presenting with DES-ISR is rising [9]. In addition, PCI for ISR has been associated with a greater risk of major adverse cardiac events when compared to PCI for de novo lesions [8, 10]. Therefore, identifying and understanding the risk factors for DES-ISR is crucial for developing strategies to prevent or mitigate this complication.

Although many studies have explored risk factors that potentially increase the incidence of DES-ISR, their findings have often been inconsistent [11, 12, 13, 14], hindering the formulation of new clinical strategies. This inconsistency, coupled with the wide range of reported risk factors and incidence rates, underscores the absence of a consensus in this area. To bridge these gaps, we conducted this systematic review and meta-analysis aimed at quantifying and summarizing both the incidence of DES-ISR and its associated risk factors. This comprehensive analysis not only elucidates the relationship between various risk factors and DES-ISR, but also provides a scientific foundation for developing preventive and management strategies tailored to patients with DES-ISR.

## 2. Materials and Methods

### 2.1 Literature Search Strategy

This protocol was registered with PROSPERO International Prospective Register of Systematic Reviews (https://www.crd.york.ac.uk/PROSPERO/display_record.php?Reco-rdID=427398, identifier: CRD42023427398) and has been reported following the Preferred Reporting Items for Systematic Reviews and Meta-Analysis Protocol (PRISMA-P) [15] and the Meta-analyses Of Observational Studies in Epidemiology (MOOSE) requirements [16].

Four databases—PubMed (MEDLINE), EMBASE, Cochrane Library, and Web of Science—were systematically searched for literature on risk factors for DES-ISR from inception to February 28, 2023. Searches were restricted to the English language. Additionally, references from recent review articles were examined to identify potentially eligible studies [9, 12, 17, 18, 19, 20, 21]. Search terms included “Drug Eluting Stent”, “DES”, “Eluting Stent”, “Eluting Coronary Stent”, “Coated Stent”, “Coated Coronary Stent”, “Coronary Restenosis”, “ISR”, “Restenosis”, “Risk”, “Cohort”, “Case-control”, combined using Boolean operators such as “AND” and “OR”. Details of search strategies are presented in the **Supplementary Materials**.

### 2.2 Study Eligibility Criteria

Articles were considered eligible for inclusion if they met the following inclusion criteria: (1) Participants were adults treated with DES; (2) Participants exposed to risk factors were compared with those not exposed to risk factors; (3) The study outcomes included DES-ISR; (4) Study types: Observational study, including cohort and case-control studies. Studies were excluded if they were: (1) Duplicate studies; (2) Lacking full text availability; (3) Studies without regression analysis examining the relationship between risk factors and DES-ISR; (4) Studies focused on clinical outcomes such as target lesion revascularization (TLR) and target vessel revascularization (TVR) or other unrelated outcomes; (5) Studies reporting associations only in specific populations at a high risk of ISR.

Two investigators independently screened all retrieved records to identify potentially eligible studies, beginning with titles and abstracts and progressing to full text reviews. Reasons for excluding studies were documented. Following independent evaluations, any discrepancies between the investigators were discussed to understand and resolve the differences. The reasons behind these differences were presented and debated within our group. If the discrepancies were resolved through discussion, the final results would be confirmed. Otherwise, a third researcher would be consulted, who independently evaluated the related research and provided his evaluation results. Subsequently, all team members discussed the third researcher’s opinions, which facilitated reaching a final consensus.

### 2.3 Data Extraction and Quality Assessment

The following information was extracted from the articles: first author, publication year, publication journal, study design, total sample size, average age, male%, average follow-up angiography time, DES type, ISR rate reported, risk factors, the value of odds ratio (OR), relative risk (RR), or hazard ratio (HR), and 95% confidence interval (CI). In case of insufficient data, an attempt was made to contact the study authors for additional data by email.

The quality of each included study was evaluated using the Newcastle-Ottawa scale (NOS) [22], a widely utilized tool for assessing the quality of cohort studies and case-control studies. This scale consists of three modules covering eight items, which include the selection of study population, comparability, and the exposure/outcome evaluation. Specifically, the selection criterion considers the representativeness of the enrolled patient sample, the comparability between the exposed and non-exposed patients, the accuracy of exposure ascertainment, and the absence of the outcome at study start. Comparability was determined based on the control of confounding factors, while exposure/outcome was determined by the objectiveness in determining the outcome and follow-duration. For instance, a study employing a random sample from multiple hospitals with comprehensive records would receive a higher score than one using a non-random sample from a single clinic. Studies are rated up to a maximum of 9 stars, with those scoring at least 6 stars considered moderate to high quality. Studies rated with less than 6 stars were excluded from our analysis.

### 2.4 Data Consolidation and Analysis

In this study, results of multivariable analysis detailing risk factors were extracted from all included studies to serve as outcomes. Risk factors reported by only one study were not subjected to pooled analysis but were described individually. For risk factors documented in two or more studies, meta-analysis was conducted using STATA software (StataCorp LLC, TX, USA). The effect sizes calculated were odds ratios (ORs) and 95% confidence intervals (CIs), applying a logit transformation for normalization. For binary variables, such as sex, where the reporting varied (e.g., male or female), consistency was achieved by uniformly converting such categories, using the reciprocal for “male” when necessary.

In a meta-analysis, if *I*^2^ < 25%, there was no heterogeneity. If *I*^2^ was between 25% and 50%, the degree of heterogeneity was considered small. If the value of *I*^2^ was between 50% to 75%, argues that there is heterogeneity; If *I*^2^ > 75%, large heterogeneity was considered. When the heterogeneity is large, the random-effects model provides more realistic assumptions when dealing with interstudy variation because it takes into account the uniqueness of each study. On the other hand, it gives the effect of the amount estimate closer to zero than the fixed effects model, so statistically more cautious. To get more reliable assumptions and more conservative results, we all selected the random effects model to summarize the effect sizes.

Sensitivity analysis is primarily used to assess if the study results are sensitive to changes in study assumptions, model choices, parameter estimates, or other key assumptions. By changing the model or parameters, researchers can test the stability and reliability of the results, and then verify the robustness of the results. In this present study, sensitivity analysis was performed by one-by-one exclusion method to evaluate the robustness of the merged results [23].

Subgroup analysis is essential for understanding how different populations respond to the same interventions, helping to explore and explain heterogeneity in study results. In this analysis, the study population was divided into subsets according to specific characteristics (such as age, sex, and disease severity), and the results of each subset are analyzed separately. In this study, subgroup analysis based on follow-up angiography time or study design was performed.

To evaluate potential publication bias, we inspected funnel plots for asymmetry in analyses that included ten or more studies. Additionally, Egger’s test was applied across all items, irrespective of the number of studies involved, to evaluate publication bias, which would be considered present if the p-value from Egger’s test was less than 0.1.

## 3. Results

### 3.1 Literature Search

**Fig. 1.**
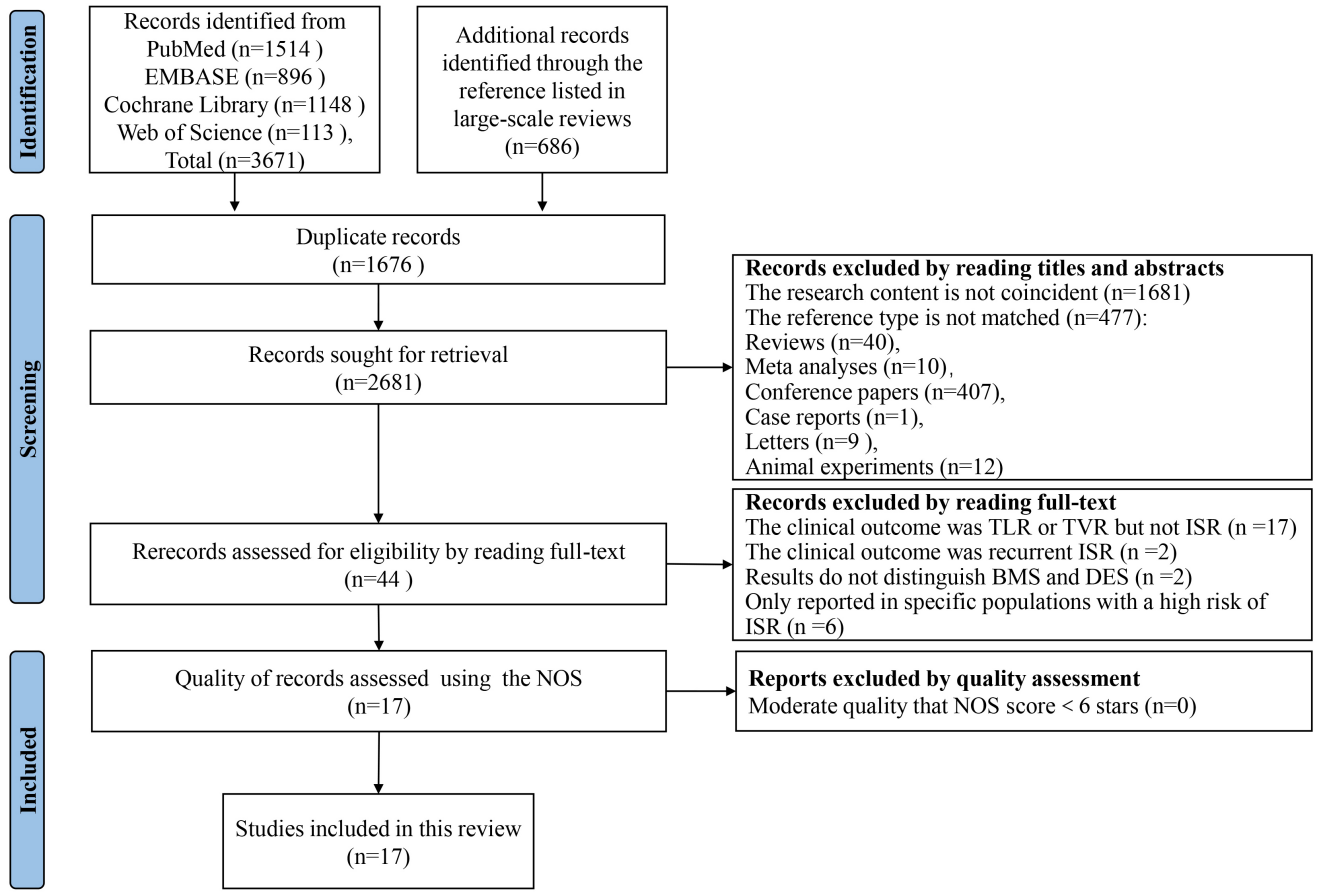
**Literature search and study selection process**. This flow diagram, structured according to the PRISMA guidelines, delineates the systematic process used to identify and screen studies for inclusion in our review and meta-analysis. Beginning with an initial retrieval of 4357 citations from four electronic databases, the figure details each step of the exclusion and inclusion process, culminating in the 17 studies that met our criteria. Each stage of the process is quantified to show the filtering of data, from initial citation count to final study selection. **Supplementary Materials** provide additional details regarding the reasons for the exclusion of specific studies. PRISMA, Preferred Reporting Items for Systematic Reviews and Meta-Analyses; NOS, Newcastle-Ottawa Scale; TLR, target lesion revascularization; TVR, target vessel revascularization; ISR, in-stent restenosis; BMS, bare metal stents; DES, drug-eluting stents.

After searching four electronic databases, we retrieved a total of 4357 citations. This collection comprised 3671 original documents along with 686 references listed in large-scale reviews. Following the removal of duplicates and the screening of titles and abstracts, 44 full-text articles were reviewed for eligibility. Ultimately, 17 studies met the inclusion criteria and were incorporated into our analysis. Within the selection, 11 are cohort studies [24, 25, 26, 27, 28, 29, 30, 31, 32, 33, 34] and 6 case-control studies [35, 36, 37, 38, 39, 40]. After quality assessment by Newcastle-Ottawa scale (NOS) scores, all studies were subsequently included in the present analysis. The process of literature search and screening is shown in Fig. 1, and all excluded records and reasons are listed in the **Supplementary Materials**.

### 3.2 Characteristics of Included Studies

Upon completion of the literature search and selection, the analysis included 17 studies of high methodological quality. These studies collectively reported on 24 risk factors assessed in two or more studies each. The characteristics of each study and the risk factors involved are shown in Table 1 (Ref. [24, 25, 26, 27, 28, 29, 30, 31, 32, 33, 34, 35, 36, 37, 38, 39, 40]), and the detailed information is shown in **Supplementary Table 1**. The mean follow-up period ranged from 6 months to 34.2 months, and the sample sizes ranged from 126 to 5355. A total of 73 risk factors were recorded across these studies, which were categorized into four main groups: patient-related, lesion-related, stent-related, and procedural factors [20] as shown in **Supplementary Table 2**. In addition, the detailed quality assessment of the included studies is provided in **Supplementary Tables 3,4**.

**Table 1.** **Characteristics and information of included studies and common risk factors involved**.

Author, year	Study design	DES subjects received re-angiography (and/or lesions number)	Age	Male (%)	Follow-up angiography time (months)	DES type	DES-ISR subjects (and/or lesions number)	Number of risk factors involved	Repeated reported risk factors	NOS scores
Hong MK, 2006 [24]	Cohort study	449 (543 lesions)	58.0 ± 10.2	71.5	6	(1)	21 (21 lesions)	2	a	8
Kastrati A, 2006 [25]	Cohort study	1495 (1703 lesions)	65.6 ± 9.6	79.0	6.43 ± 2.10	(1) (2)	(222 lesions)	3	b	8
Park D-W, 2007 [26]	Cohort study	1172	61.3 ± 10.3	71.3	6	(1) (2)	125	3	c, d	8
Roy P, 2007 [35]	Case-control study	3535 (5046 lesions)	65.4 ± 11.6	65.2	12	(1) (2)	197 (237 lesions)	15	b, e, f, g, h, i, j, k	7
Kitahara H, 2009 [36]	Case-control study	1312 lesions	67.3 ± 9.47	81.5	6–9	(1)	122 (124 lesions)	5	a, f, g, l	6
Ino Y, 2011 [37]	Case-control study	399 (537 lesions)	68.0 ± 9.8	79.4	6–9	(1)	37 (44 lesions)	10	a, c, d, f, m	7
Kim YG, 2013 [38]	Case-control study	1069	64.5 ± 10.05	69.3	6–9	(1) (2) (3)	119 (161 lesions)	11	a, f, g, k, n, o	6
Cassese S, 2014 [27]	Cohort study	5355 (8483 lesions)	65.4 ± 12.3	75.6	6–8	(1) (2) (3) (4)	(1130 lesions)	11	a, f, i, p	6
Park SH, 2015 [28]	Cohort study	439 (683 lesions)	63.5 ± 9.6	65.2	6–9	(1) (2) (3)	(69 lesions)	12	c, e, f, g, h, l, m, n, o, p, q	7
Zhao L-P, 2015 [29]	Cohort study	417	65.0 ± 11.5	77	17.5 ± 10.2	(1) (3)	58	3	p, r	7
Gabbasov Z, 2018 [30]	Cohort study	126	62.3 ± 10.6	75.4	6–12	-	53	5	f, j	7
XU X, 2019 [39]	Case-control study	612	62.3 ± 9.1	77.9	6–24	-	95	7	e, f, l, n, o, s	6
Gai M-T, 2021 [31]	Cohort study	986	59.0 ± 10.8	78.5	16.93	-	56	6	q, t, u	6
Gupta PK, 2021 [32]	Cohort study	550	54.3 ± 9.4	85.3	24.37 ± 9.18	(1) (2) (3) (4)	31	7	a, f, i, j, p	6
Zhu Y, 2021 [33]	Cohort study	1574	58.4 ± 9.4	77.4	12	(1) (3) (4)	253	16	a, b, e, f, g, i, r, n, v, w, x	6
Lin XL, 2022 [34]	Cohort study	797	59.0 ± 9.6	75.3	6	(1) (3) (4)	202	16	a, e, f, j, p, q, r, s, u, v, w, x	6
Li M, 2022 [40]	Case-control study	341	65.8 ± 10.9	63.0	34.2 ± 17.2	(1) (3) (4)	62	6	a, b, j, q, t, v	6

(1) SES; (2) PES; (3) ZES; (4) EES. a, stent length (mm); b, SES; c, postintervention MLD (mm); d, stents per lesion (n); e, age; f, DM; g, hypertension; h, dyslipidemia; i, LAD; j, number of stents; k, stent diameter; l, lesion length; m, RVD; n, sex; o, smoking; p, multivessel disease; q, LDL-C; r, BMI; s, multiple stents; t, TC; u, medical history of MI; v, LVEF; w, previous PCI; x, minimal stent diameter. SES, sirolimus-eluting stents; PES, paclitaxel-eluting stents; ZES, zotarolimus-eluting stent; EES, everolimus-eluting stents; MLD, minimal luminal diameter; DM, diabetes mellitus; LAD, left anterior descending artery; RVD, reference vessel diameter; LDL-C, low-density lipoprotein cholesterol; BMI, body mass index; TC, total cholesterol; MI, myocardial infarction; LVEF, left ventricular ejection fraction; PCI, percutaneous coronary intervention; DES, drug-elutingstents; ISR, in-stent restenosis; NOS, Newcastle-Ottawa Scale.

### 3.3 Meta-Analysis

#### 3.3.1 Incidence of DES-ISR

**Fig. 2.**
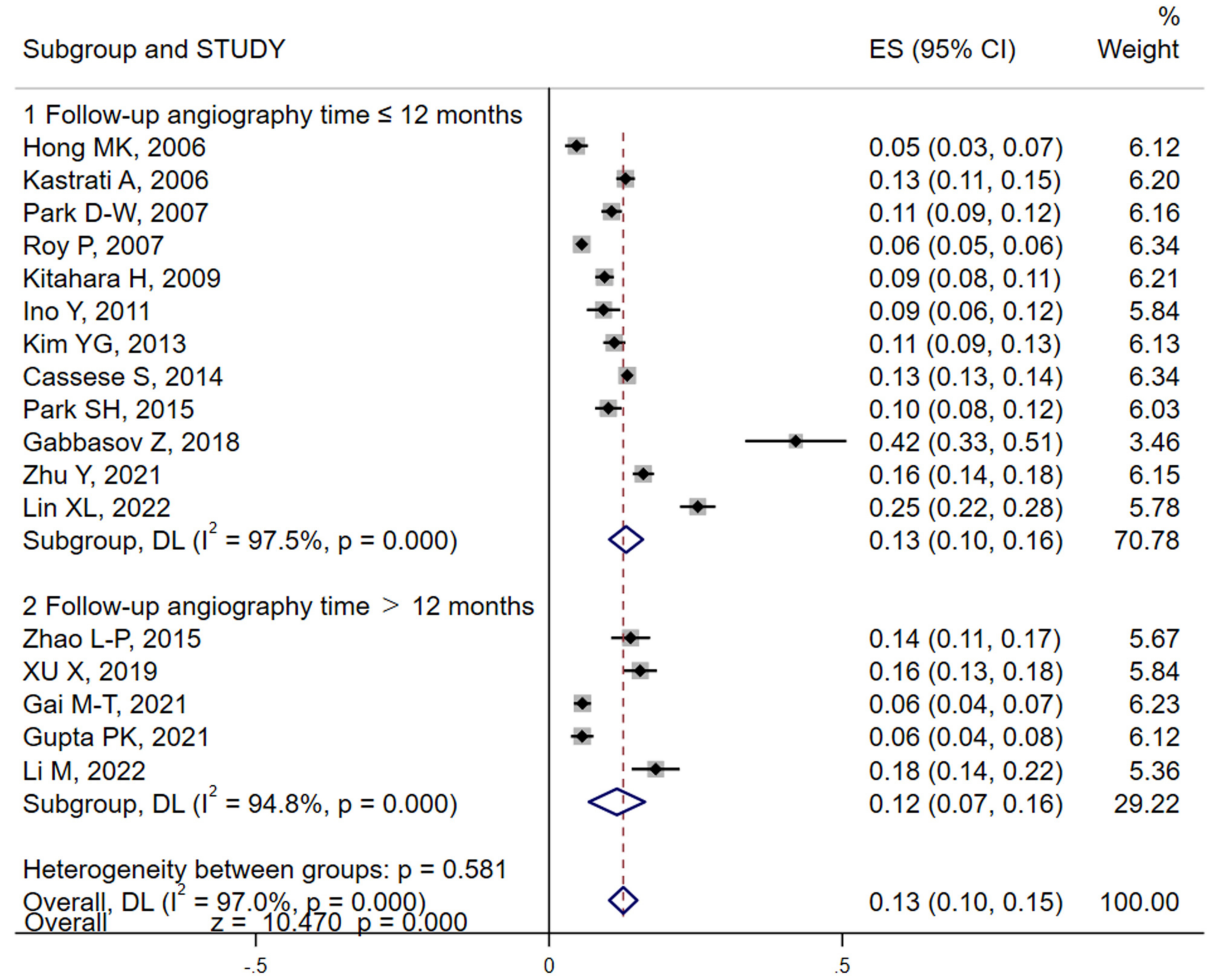
**Analysis of DES-ISR incidence and by follow-up duration**. This forest plot visualizes the pooled incidence rates of ISR among patients with DES across different follow-up periods. The analysis distinguishes between shorter and longer follow-up durations to assess variations in ISR rates over time. The plot includes individual study results with their respective confidence intervals, highlighting the overall pooled estimate using a random-effects model to account for study heterogeneity. Subgroup analyses are also depicted to further explore how follow-up time impacts ISR rates. DES, drug-eluting stents; ISR, in-stent restenosis; ES, effect size; DL, DerSimonian-Laird.

In the studies we analyzed, both patient and lesion-based incidences of ISR were reported. We primarily utilized the number of subjects as our main variable for the meta-analysis. However, lesion data were also included when patient data were incomplete. Our meta-analysis, conducted using random effects models, revealed that the pooled result (Fig. 2) of ISR for DES was approximately 13% (95% CI: 10%–15%) by using random effects models, albeit with significant heterogeneity (*I*^2^ = 97.0%). Sensitivity analyses were performed employing a one-by-one elimination to locate, the source of heterogeneity (Gabbasov *et al*., [30]). Removal of this study from the analysis resulted in a similar incidence rate, of 12% (95% CI: 9%–14%), confirming robustness of our results (**Supplementary Fig. 1A**). Further subgroup analyses using follow-up angiography time and study design as variables show no significant changes in the results between groups (Fig. 2 and **Supplementary Fig. 1B**).

#### 3.3.2 Risk factors of DES-ISR 

**Fig. 3.**
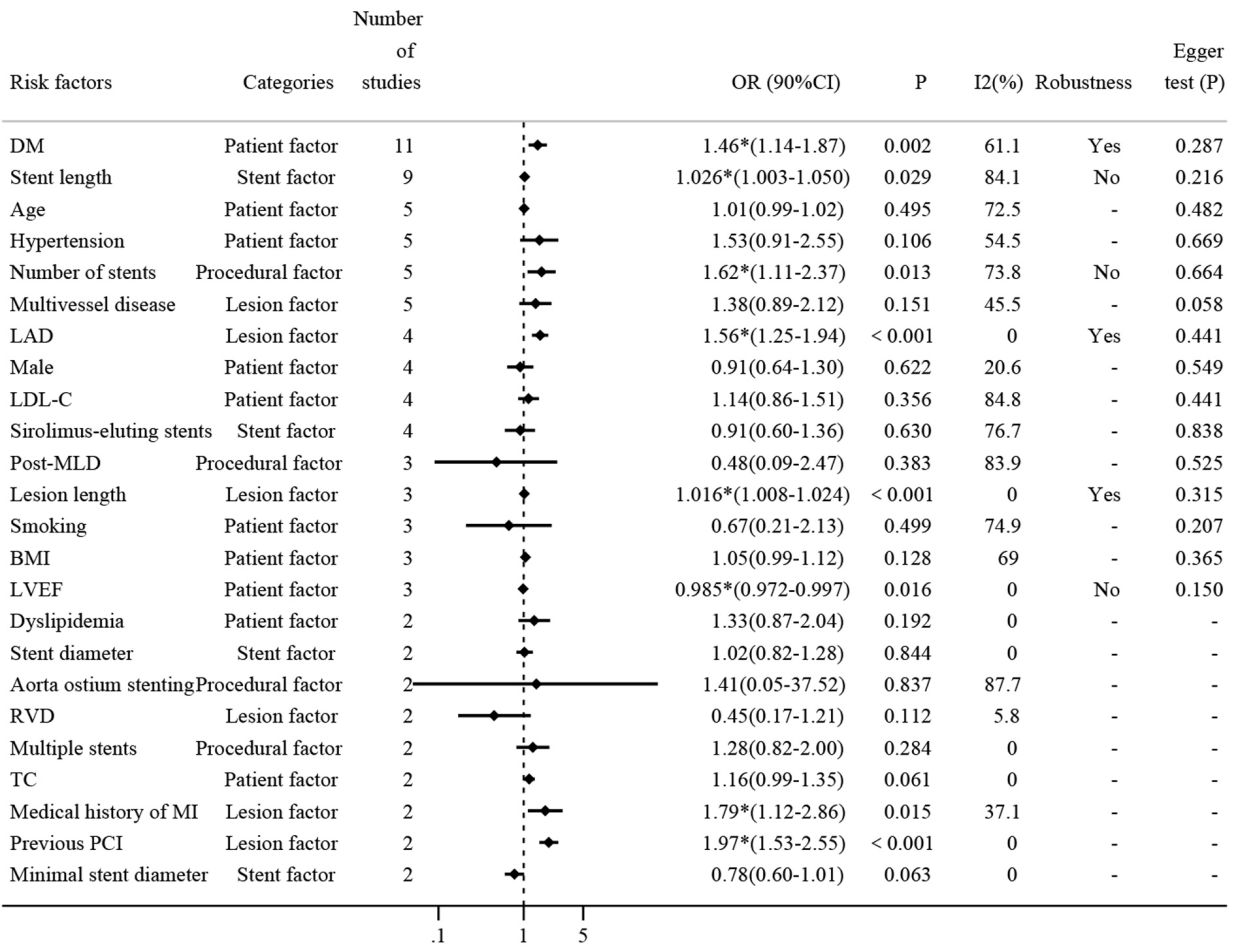
**Comprehensive analysis of risk factors for DES-ISR**. Fig. 3 presents a forest plot summarizing the ORs and 95% CIs for repeatedly reported risk factors associated with DES-ISR. Each line represents a different study’s findings for the respective risk factor, highlighting their impact on ISR occurrence and providing a visual representation of the pooled effect sizes calculated using a random-effects model. DM, diabetes mellitus; LAD, left anterior descending artery; RVD, reference vessel diameter; LDL-C, low-density lipoprotein cholesterol; BMI, body mass index; TC, total cholesterol; MI, myocardial infarction; LVEF, left ventricular ejection fraction; PCI, percutaneous coronary intervention; DES, drug-eluting stents; ISR, in-stent restenosis; MLD, minimal luminal diameter. * means *p* < 0.05, and the combined effect of risk factor is statistically significant.

To systematically identify the impact of various risk factors on the incidence of ISR, a detailed meta-analysis was performed on 24 risk factors, each reported in at least two of the 17 included studies. The results of all risk factors are summarized in Fig. 3, highlighting eight factors that showed statistically significant results, which include seven risk factors and one protective factor.

Firstly, diabetes mellitus (DM) was reported in 11 studies with approximately 15,769 patients. The pooled results show that diabetes increased the risk of ISR by 46% (OR: 1.46, 95% CI: 1.14–1.87, **Supplementary Fig. 2**). The heterogeneity test shows that *I*^2^ = 61.1% > 50%, indicating significant heterogeneity among the studies. Subgroup analysis by study type showed that there was no significant heterogeneity in the cohort study group, however, considerable heterogeneity persisted among the case-control studies, likely due to their smaller sample sizes. This heterogeneity was not a determinant of the type of study, indicating other underlying factors. Sensitivity analysis carried out through the one-by-one omission method affirmed the stability of the pooled results (95% CI does not include 1.00), indicating the robustness of the present analysis (**Supplementary Fig. 3**). Additionally, the funnel plot and Egger test were used to check the publication bias. The distribution of the studies in the funnel plot was roughly symmetrical (**Supplementary Fig. 4**), and the Egger test displays *p* = 0.287 > 0.05, suggesting there was no publication bias.

The second most reported risk factor was stent length (mm), which was discussed in nine studies. The pooled analysis demonstrated that each unit increase in stent length contributed to a 3% increase in DES-ISR (OR: 1.03, 95% CI: 1.00–1.05, **Supplementary Fig. 5**), although this result was marked by significant heterogeneity. Subgroup analysis showed the type of study did not contribute to this heterogeneity. Further sensitivity analysis revealed that the meta-analysis lacked robustness, potentially due to the influence of the study by Hong MK [24], where the confidence interval included 1.00, suggesting it as a primary source of the observed heterogeneity (**Supplementary Fig. 6**). Additionally, Egger’s test indicated no evidence of publication bias (*p* = 0.216 > 0.05).

Several other risk factors were investigated for their association with DES-ISR in five different studies (**Supplementary Fig. 7**). Age, hypertension, number of stents, and multivessel disease were all reported in five studies, and pooled results showed that only the number of stents was a statistically significant risk factor (OR: 1.62, 95% CI: 1.11–2.37). Sensitivity analysis indicated that the heterogeneity may result from studies conducted by Hong MK [24] and Kitahara [36], which even can be considered as a main source of significant heterogeneity (**Supplementary Fig. 8**). Additionally, there was no publication bias as determined by Egger’s test (*p* = 0.664 > 0.05).

Among the diverse range of risk factors evaluated, the Left anterior descending artery (LAD), sex, low-density lipoprotein cholesterol (LDL-C), and sirolimus-eluting stents were all reported in four studies (**Supplementary Fig. 9**), Pooled results showed that only LAD was significantly associated with DES-ISR (OR: 1.56, 95% CI: 1.25–1.94). Sensitivity analysis indicated that the original meta-analysis has good robustness (**Supplementary Fig. 10**). Again, there was no publication bias as determined by Egger’s test (*p* = 0.441 > 0.05).

To further elucidate the impact of various clinical and procedural variables on DES-ISR, our meta-analysis included studies that reported on post-minimal luminal diameter (MLD), lesion length, smoking, body mass index (BMI), and left ventricular ejection fraction (LVEF), each addressed in three separate studies (**Supplementary Fig. 11**). The meta-analysis revealed that lesion length significantly contributes to the risk of ISR (OR: 1.016; 95% CI: 1.008–1.024). Sensitivity analysis confirmed the robustness of these findings (**Supplementary Fig. 12**), with no evidence of publication bias as indicated by Egger’s test (*p* = 0.315 > 0.05). Additionally, LVEF was identified as a statistically significant protective factor (OR: 0.98, 95% CI: 0.97–1.00), though sensitivity analysis suggested that the results for this variable lacked robustness (**Supplementary Fig. 13**). No publication bias was detected by Egger’s test (*p* = 0.150 > 0.05).

Several clinical and procedural variables were each reported in two studies, as detailed in **Supplementary Fig. 14**. These variables include dyslipidemia, stent diameter, aorta ostium stenting, reference vessel diameter (RVD), multiple stents, total cholesterol (TC), medical history of myocardial infarction (MI), previous percutaneous coronary intervention (PCI), and minimal stent diameter, and were each reported twice. Notably, a medical history of MI and previous PCI were identified as significant risk factors for DES-ISR (OR: 1.79; 95% CI: 1.12–2.86; and OR: 1.97, 95% CI: 1.53–2.55 respectively). Due to the limited number of studies (two), sensitivity analysis and assessment of publication bias were not conducted for these factors.

## 4. Discussion

In our comprehensive systematic review and meta-analysis, we meticulously evaluated the incidence and risk factors associated with DES-ISR. Despite advancements in stent technology, ISR continues to pose a substantial challenge, occurring at an approximate rate of 13% even in the modern era of DES. This rate significantly impacts both the effectiveness of stent therapy and the long-term outcomes for patients. Our analysis confirmed that DM, stent length, number of stents, involvement of the LAD, lesion length, medical history of MI, and previous PCI are significant risk factors for DES-ISR. Conversely, a higher LVEF was identified as a protective factor. However, the potential influence of other factors such as age, hypertension, multivessel disease, male sex, LDL-C, sirolimus-eluting stents, post-MLD, smoking, BMI, dyslipidemia, stent diameter, aorta ostium stenting, RVD, multiple stents, TC, and minimal stent diameter on ISR remains unclear due to the lack of statistically significant associations. These factors warrant further investigation to fully elucidate their roles in the pathogenesis of ISR.

The notable incidence of DES-ISR rate observed in the present study, approximately 13%, is supported by previous research reporting DES-ISR rates exceeding 10% in unselected patients [27]. This finding underscores the necessity for ongoing surveillance and the development of more effective strategies to reduce the incidence of recurrent DES-ISR.

While ISR significantly impacts patient outcomes by often leading to the recurrence of angina symptoms or an acute coronary syndrome, requiring repeated revascularization therapy [41], the underlying mechanisms are complex. The initial vascular endothelial injury caused by stent implantation triggers a cascade of inflammatory responses that promote the proliferation, migration, and differentiation of vascular smooth muscle cells, a predominant pathological process [17]. Although DES release anti-inflammatory, immunomodulatory, or antiproliferative agents such as sirolimus, paclitaxel, and everolimus, which effectively inhibit intimal hyperplasia and greatly reduce the incidence of restenosis [42], ISR remains a concern. This persistence of ISR despite advances in stent technology suggests a need for deeper investigation into its mechanisms and specific risk factors. The incidence of ISR has been proven multifactorial, and a variety of factors can affect the development of ISR, including patient-specific factors, lesion characteristics, stent design, and procedural details [20, 43].

In terms of patient factors, consistent with our findings, previous studies have identified DM, medical history of MI, and previous PCI as risk factors for DES-ISR [31, 33, 34]. Conversely, we found LVEF to be a protective factor.

Multiple studies have established that patients with DM are at a higher risk of developing DES-ISR [3, 44, 45]. Several potential mechanisms are implicated in this increased risk including inflammation, hypercoagulability, alterations in blood rheology, endothelial dysfunction, and excess neointimal hyperplasia associated with DM [46]. One contributing factor is chronic oxidative stress, driven by elevated glucose levels and the production of advanced glycation end-products (AGEs), which damage endothelial cells lining the arterial walls, leading to dysfunction and increased inflammation [47]. This chronic inflammation can lead to an overactive immune response that contributes to the development of atherosclerotic plaques and restenosis [48].

In addition, the proliferation of vascular smooth muscle cells (VSMCs) in the arterial walls increases under DM conditions, spurred by glucose-induced activation of signaling pathways that enhance cell proliferation and migration [49]. Meanwhile, DM promotes abnormal vascular remodeling, characterized by increased intima-media thickness and the development of fibrotic plaques, predisposing arteries to restenosis [50]. In diabetic patients, this fibrotic response is often exacerbated, with increased deposition of extracellular matrix components like collagen, leading to arterial narrowing and plaque formation [51].

DM also can lead to hypercoagulability, heightening thrombosis risk, which can lead to the formation of thrombotic occlusions that can inhibit the healing process and contribute to restenosis [52]. In this context, compromised endothelial function, further deteriorates arterial health, weakening the healing response after stent implantation, increasing the risk of restenosis [53]. Collectively, these factors increase the risk of new atherosclerosis and DES-ISR [54]. This underscores the importance of meticulous follow-up and targeted management strategies for diabetic patients who undergo DES implantation.

A medical history of MI and previous PCI typically indicates more severe and complex lesions. Injuries from previous procedures can increase the likelihood of DES-ISR, and may contribute to drug resistance, which may also play a role in the mechanism behind ISR [43]. Inflammation triggered by MI or prior PCI procedures can lead to excessive proliferation of in-stent tissue, while vascular endothelial injury hampers the repair process of the vessel wall [55]. Additionally, vascular remodeling processes, including wall thickening, smooth muscle cell proliferation, and fibrosis, all are possible mechanism of increased risk of ISR. Furthermore, a decreased LVEF means impaired left ventricular function that predicts a poor prognosis [56]. Our findings suggest that the lower LVEF correlates with a higher the incidence of DES-ISR. While the direct mechanism linking cardiac function and ISR remains unclear, this association underscores the importance of routine postoperative echocardiography to reassess cardiac function following stent implantation.

In terms of lesion factors, our findings, along with previous research, demonstrate that both LAD and lesion length significantly elevate the risk of ISR. Due to the unique anatomical characteristics of the left main coronary artery, lesions associated with LAD are often complex, involving multiple vessel disease (MVD) or ostial lesions [57]. Despite technological advancements and numerous clinical trials showing DES to significantly decrease revascularization rates in LAD lesions when compared to historic single-vessel bypass surgery [58], the incidence of ISR remains comparatively high in the LAD. In this study, we found that LAD lesions could increase the occurrence of ISR, which is consistent with previous studies that have confirmed the rate of ISR in the LAD is significantly higher when compared to the circumflex branch and the right coronary artery [59, 60].

Several anatomical and physiological factors contribute to this increased risk. Vessel size and lesion involvement the LAD typically has a larger diameter and involves a major portion of the vessel, which may lead to inadequate vascular remodeling post-stent implantation, subsequently increasing the risk of DES-ISR [61]. Kinking and bifurcation: areas of kinking and bifurcation within the LAD can compromise stent adhesion, impairing stent expansion and vessel wall healing, thereby increasing the risk of DES-ISR [62]. Blood Flow Dynamics: the LAD region experiences a higher blood flow velocity and shear stress, which may lead to vascular endothelial damage, thereby contributing to the development of DES-ISR [63, 64]. Surgical challenges: the anatomical positioning and vascular conditions of LAD can complicate stent implantation, often necessitating specialized techniques or equipment, which may affect the surgical procedure and postoperative repair [63, 64].

Multiple factors contribute to the complexity of managing ISR. Notably, lesions that are too long may not be adequately covered by stents, leading to geographic loss [65], a recognized risk factor in the occurrence of ISR. Longer lesions also provide a larger source of smooth muscle cells, which proliferate and form neointima, exacerbating restenosis [66]. Additionally, the risk of restenosis increased with stent length, a finding consistent with prior research [67, 68]. While using longer stents to ensure full lesion coverage is the preferred strategy in PCI [69], it paradoxically also heightens the risk of restenosis. In addition, more studies are focusing on the ratio of stent to lesion length and focusing stent placement at the primary obstruction site to minimize restenosis risk [35, 70]. Procedurally, the use of multiple stents is linked to greater vascular damage and a subsequent increase in restenosis risk [71]. This damage often triggers inflammation, promoting the proliferation of fibroblasts and smooth muscle cells, which contribute to the development of restenosis [71].

The present study has several limitations to be noted. First, the inherent heterogeneity among the original studies and the variable quality of the databases used for meta-analysis could introduce bias, potentially leading to an overestimation or underestimation of the overall results. Secondly, our pooled multivariable data from all included studies, which may vary significantly in terms of the number of predictors, granularity, and the handling of missing values, as well as the number of patients and events. These discrepancies underscore the need for further targeted investigations. In addition, coronary artery disease complexity is an important factor that impacts disease incidence and the assessment of the effect size of risk factors. However, when we reviewed the angiographic data across the studies, we found that the original studies did not provide sufficient data to differentiate between the complexity of coronary artery disease for analysis. Finally, some risk factors such as age, sex, hypertension, and smoking did not reach statistical significance in our meta-analysis, but have been frequently reported in studies and reviews and should not be ignored. Further research is required to confirm our findings.

## 5. Conclusions

While DES have significantly mitigated the occurrence of ISR, the incidence remains at approximately 13% in current clinical populations. Our meta-analysis identified DM, stent length, number of stents, LAD involvement, lesion length, medical history of MI, and previous PCI as primary risk factors for DES-ISR. Conversely, a higher LVEF was highlighted as a protective factor. Understanding these risk factors is crucial for developing a predictive model for DES-ISR, which can significantly inform clinical practices and enhance postoperative long-term care strategies. However, to refine these models and further improve patient outcomes, there is an urgent need for larger, higher-quality clinical trials that can provide more definitive evidence and clearer guidance for managing patients with DES.

## Data Availability

Data availability is not applicable to this article as no new data were created or analyzed in this study and most of the data were obtained from the references in this article.

## Abbreviations

DES, drug-eluting stents; ISR, in-stent restenosis; BMS, bare metal stents; OR, odds ratio; PCI, percutaneous coronary intervention; CAD, coronary artery disease; DCB, drug-coated balloons; BRS, bioresorbable scaffolds; PRISMA-P, Preferred Reporting Items for Systematic Reviews and Meta-Analysis Protocol; MOOSE, Meta-analyses Of Observational Studies in Epidemiology; TLR, target lesion revascularization; TVR, target vessel revascularization; RR, relative risk; HR, hazard ratio; CI, confidence interval; NOS, Newcastle-Ottawa scale; SES, sirolimus-eluting stents; PES, paclitaxel-eluting stents; ZES, zotarolimus-eluting stent; EES, Everolimus-eluting stents; MLD, minimal luminal diameter; DM, diabetes mellitus; LAD, left anterior descending artery; RVD, reference vessel diameter; LDL-C, low-density lipoprotein cholesterol; BMI, body mass index; TC, total cholesterol; MI, myocardial infarction; LVEF, left ventricular ejection fraction; AGEs, advanced glycation end-products; VSMCs, vascular smooth muscle cells.

## Supplementary Material

Supplementary material associated with this article can be found, in the online version, at https://doi.org/10.31083/j.rcm2512458.
