# Supplementary material for: Risk Factors and Incidence for In-Stent Restenosis with Drug-Eluting Stent: A Systematic Review and Meta-Analysis
Source: Rev Cardiovasc Med. 2024 Dec 24;25(12):458. doi: 10.31083/j.rcm2512458 (PMC11683691; doi:10.31083/j.rcm2512458)
Supplement: Supplementary file 1 [file 2153-8174-25-12-458-s1.zip › RCM23196-Supplementary Material-V2.pdf]

Supplementary Materials

This PDF file includes:

1. The search strategy of PubMed
2. Excluded records
3. Supplementary Tables 1 to 4
4. Supplementary Fig. 1-14
5. MOOSE (Meta-analyses Of Observational Studies in Epidemiology) Checklist
6. NEWCASTLE - OTTAWA QUALITY ASSESSMENT SCALE

1. The search strategy of PubMed

1. "Drug Eluting Stent*"[Mesh]
2. DES [Title/Abstract]
3. Eluting Stent*[Title/Abstract]
4. Eluting Coronary Stent*[Title/Abstract]
5. Coated Stent*[Title/Abstract]
6. Coated Coronary Stent*[Title/Abstract]
7. 1 OR 2 OR 3 OR 4 OR 5 OR 6
8. "Coronary Restenosis"[Mesh]
9. ISR[Title/Abstract]
10. Restenosis [Title/Abstract]
11. 8 OR 9 OR 10
12. Risk*[Title/Abstract]
13. Cohort [Title/Abstract]
14. Case-control [Title/Abstract]
15. 12 OR 13 OR 14
16. 7 AND 11 AND 15

2. Excluded records

2.1 The clinical outcome was TLR or TVR but not ISR (n =17)

[1] Otsuka Y, Ishiwata S, Inada T, et al. Comparison of haemodialysis patients and non-haemodialysis patients with respect to clinical characteristics and 3-year clinical outcomes after sirolimus-eluting stent implantation: insights from the Japan multi-centre post-marketing surveillance registry. Eur Heart J. 2011;32:829-837.

[2] Tahara S, Bezerra HG, Kyono H, et al. Impact of Acute Gain on Clinical Outcomes of Patients Treated With Sirolimus-Eluting Stent - A Sub-Analysis Study From the STLLR Trial. Circulation Journal. 2011;75:2113-2119.

[3] Lemos PA, Arampatzis CA, Hoye A, et al. Impact of baseline renal function on mortality after percutaneous coronary intervention with sirolimus-eluting stents or bare metal stents. Am J Cardiol. 2005;95:167-172.

[4] Kedhi E, Généreux P, Palmerini T, et al. Impact of coronary lesion complexity on drug-eluting stent outcomes in patients with and without diabetes mellitus: analysis from 18 pooled randomized trials. J Am Coll Cardiol. 2014;63:2111-2118.

[5] Kurihara K, Ashikaga T, Sasaoka T, Yoshikawa S, Isobe M. Incidence and predictors of early and late target lesion revascularization after everolimus-eluting stent implantation in unselected patients in japan. Catheter Cardiovasc Interv. 2017;90:78-86.

[6] Abdel-Wahab M, Neumann FJ, Serruys P, et al. Incidence and predictors of unplanned non-target lesion revascularisation up to three years after drug-eluting stent implantation: insights from a pooled analysis of the RESOLUTE Global Clinical Trial Program. EuroIntervention. 2016;12:465-472.

[7] Stolker JM, Kennedy KF, Lindsey JB, et al. Predicting Restenosis of Drug-Eluting Stents Placed in Real-World Clinical Practice Derivation and Validation of a Risk Model From the EVENT Registry. Circulation-Cardiovascular Interventions. 2010;3:327-334.a

[8] Tadano Y, Kotani JI, Kashima Y, et al. Predictors of clinical outcomes after coronary implantation of bioresorbable polymer sirolimus-eluting Ultimaster stents in all-comers: A report of 1,727 cases. Catheter Cardiovasc Interv. 2019;94:91-97.

[9] Hong M-K, Mintz GS, Lee CW, et al. Late target lesion revascularization after implantation of sirolimus-eluting stent. Catheterization and Cardiovascular Interventions. 2008;71:299-303.

[10] Elezi S, Dibra A, Mehilli J, et al. Vessel size and outcome after coronary drug-eluting stent

placement: Results from a large cohort of patients treated with sirolimus- or paclitaxel-eluting stents. Journal of the American College of Cardiology. 2006;48:1304-1309.
[11] Zahn R, Hamm CW, Schneider S, et al. Incidence and predictors of target vessel revascularization and clinical event rates of the sirolimus-eluting coronary stent (results from the prospective multicenter German Cypher Stent Registry). American Journal of Cardiology. 2005;95:1302-1308.
[12] Costa MA, Angiolillo DJ, Tannenbaum M, et al. Impact of Stent Deployment Procedural Factors on Long-Term Effectiveness and Safety of Sirolimus-Eluting Stents (Final Results of the Multicenter Prospective STLLR Trial). The American Journal of Cardiology. 2008;101:1704-1711.
[13] Choi IJ, Koh YS, Lim S, et al. Impact of the stent length on long-term clinical outcomes following newer-generation drug-eluting stent implantation. Am J Cardiol. 2014;113:457-464.
[14] Zheng C, Kang J, Park KW, et al. The Predictors of Target Lesion Revascularization and Rate of In-Stent Restenosis in the Second-Generation Drug-Eluting Stent Era. J Interv Cardiol. 2019;2019:3270132.
[15] Solinas E, Nikolsky E, Lansky AJ, et al. Gender-specific outcomes after sirolimus-eluting stent implantation. J Am Coll Cardiol. 2007;50:2111-2116.
[16] Sangiorgi G, Romagnoli E, Biondi-Zoccai G, et al. Percutaneous coronary implantation of sirolimus-eluting stents in unselected patients and lesions: Clinical results and multiple outcome predictors. American Heart Journal. 2008;156:871-878.
[17] Zahn R, Hamm CW, Schneider S, et al. Incidence and predictors of target vessel revascularization and clinical event rates of the sirolimus-eluting coronary stent (results from the prospective multicenter German Cypher Stent Registry). American Journal of Cardiology. 2005;95:1302-1308.

2.2 The clinical outcome was recurrent ISR (n =2)

[1] Rathore S, Kinoshita Y, Terashima M, et al. A comparison of clinical presentations, angiographic patterns and outcomes of in-stent restenosis between bare metal stents and drug eluting stents. EuroIntervention. 2010;5:841-846.
[2] Fujii K, Mintz GS, Kobayashi Y, et al. Contribution of stent underexpansion to recurrence after sirolimus-eluting stent implantation for in-stent restenosis. Circulation. 2004;109:1085-1088.

2.3 Results do not distinguish BMS and DES (n =2)

[1] Kalyoncuoglu M, Ozkan A, Kaya A, Yuksel Y, Dogan N, Gurmen A. A new predictor of in-stent restenosis in patients undergoing elective percutaneous coronary İntervention: triglyceride glucose İndex. International Journal of the Cardiovascular Academy. 2021;7:50-54.
[2] Baktashian M, Soflaei SS, Kosari N, et al. Association of high level of hs-CRP with in-stent restenosis: A case-control study. Cardiovascular Revascularization Medicine. 2019;20:583-587.

2.4 Only reported in specific populations with a high risk of ISR (n =6)

[1] Frobert O, Lagerqvist B, Carlsson J, Lindback J, Stenestrand U, James SK. Differences in Restenosis Rate With Different Drug-Eluting Stents in Patients With and Without Diabetes Mellitus A Report From the SCAAR (Swedish Angiography and Angioplasty Registry). Journal of the American College of Cardiology. 2009;53:1660-1667.
[2] Tiroch K, Mehilli J, Byrne RA, et al. Impact of coronary anatomy and stenting technique on long-term outcome after drug-eluting stent implantation for unprotected left main coronary artery disease. JACC Cardiovasc Interv. 2014;7:29-36.
[3] Sugita H, Motohiro M, Morishita S, Tanaka M, Tsujimoto S, Shiojima I. Factors Associated with Coronary In-Stent Restenosis after Drug-Eluting Stent Implantation in Patients on Chronic Hemodialysis. Blood Purif. 2022;51:383-389.
[4] Park HJ, Seo SM, Shin WS, et al. Soluble receptor for advanced glycation end products is associated with in-stent restenosis in patients with type 2 diabetes with drug-eluting coronary stents.

Coron Artery Dis. 2011;22:12-17.
[5] Camaj A, Giustino G, Claessen BE, et al. Effect of stent diameter in women undergoing percutaneous coronary intervention with early- and new-generation drug-eluting stents: From the WIN-DES collaboration. Int J Cardiol. 2019;287:59-61.
[6] Lee CW, Suh J, Lee SW, et al. Factors predictive of cardiac events and restenosis after sirolimus-eluting stent implantation in small coronary arteries. Catheter Cardiovasc Interv. 2007;69:821-825.

Supplementary Table 1. Detailed information of the risk factors in included studies.

Author, year	Risk factors involved
Hong MK, 2006	01, 02
Kastrati A, 2006	03, 04, 05
Park D-W, 2007	06, 07, 08
Roy P, 2007	04, 09, 10, 11, 12, 13, 14, 15, 16, 17, 18, 19, 20, 21, 22
Kitahara H, 2009	01, 10, 11, 23, 24
Ino Y, 2011	01, 07, 08, 10, 25, 26, 27, 28, 29, 30
Kim YG, 2013	01, 10, 11, 20, 26, 31, 32, 33, 34, 35, 36
Cassese S, 2014	01, 10, 14, 37, 38, 39, 40, 41, 42, 43, 44
Park SH, 2015	07, 09, 10, 11, 12, 24, 30, 31, 32, 37, 45, 46
Zhao L-P, 2015	37, 47, 48
Gabbasov Z, 2018	10, 18, 49, 50, 51
XU X, 2019	09, 10, 24, 31, 32, 52, 53
Gai M-T, 2021	45, 54, 55, 56, 57, 58
Gupta PK, 2021	01, 10, 14, 18, 37, 59, 60
Zhu Y, 2021	01, 04, 09, 10, 11, 14, 47, 61, 62, 63, 64, 65, 66, 67, 68, 69
Lin XL, 2022	01, 09, 10, 18, 37, 45, 47, 52, 56, 63, 65, 69, 70, 71, 72, 73
Li M, 2022	01, 04, 18, 45, 54, 63

01. stent length; 02. final minimum stent CSA; 03. 0.5-mm decrease in vessel size; 04. SES; 05. 5% increase in final diameter stenosis; 06. PES; 07. postintervention MLD; 08. stents per lesion (n); 09. age; 10.DM; 11. hypertension; 12. dyslipidemia; 13. unstable angina pectoris; 14. LAD; 15. ISR lesion; 16. length of procedure; 17. number of lesions treated; 18. number of stents; 19. stented length/lesion; 20. stent diameter; 21. IVUS guidance; 22. glycoprotein IIb/IIIa inhibitor use; 23. chronic renal failure; 24. lesion length; 25. hemodialysis; 26. aorta ostium stenting; 27. Δangle (every 1-degree increment); 28. stent overlap; 29. maximal angle (every 1-degree increment); 30. RVD; 31. male; 32. smoking; 33. creatinine; 34. perforation during procedure; 35. edge calcification; 36. edge maximal angulation (every 1° increment); 37. multivessel disease; 38. history of by-pass surgery; 39. NSTEMI; 40. complex lesion; 41. chronic occlusion; 42. vessel size (for 0.5 mm reduction); 43. stenosis severity (for 5% diameter stenosis increase); 44. ballon-to-vessel ratio (for 0.1unit increase); 45. LDL-C; 46. Lp(a)≥50mg/dl; 47. BMI; 48. IRI; 49. CD45+ platelet; 50. neutrophil to lymphocyte ratio; 51. arterial diameter ≤2.75 m, RLP-C; 54. TC; 55. SBP; 56. medical history of MI; 57. PDW; 58. lesion vessels; 59. vessel Calcification; 60. complications during PCI; 61. TyG index; 62. female; 63. LVEF; 64. hs-CRP; 65. Previous PCI; 66. SYNTAX score; 67. RCA; 68. intracoronary image; 69. minimal stent diameter; 70. GA; 71. FBG; 72. HbA1c; 73. one vessel disease. CSA, cross-sectional area; SES, sirolimus-eluting stents; PES, paclitaxel-eluting stent; MLD, minimal luminal diameter; DM, diabetes mellitus; LAD, left anterior descending artery (LAD); ISR, in-stent restenosis; IVUS, intravascular ultrasound; RVD, reference vessel diameter; NSTEMI, Non-ST-segment myocardial infarction; LDL-C, low-density lipoprotein cholesterol; Lp (a), lipoprotein (a); BMI, body mass index; IRI, insulin resistance index; RLP-C, remnant lipoprotein cholesterol; TC, total cholesterol; SBP, systolic blood pressure (SBP); MI, myocardial infarction ; PWV, platelet distribution width; PCI, percutaneous coronary intervention; 61. TyG, triglyceride and glucose; LVEF, left ventricular ejection fraction; hs-CRP, high-sensitivity C-reactive protein; SYNTAX, SYNergy between percutaneous coronary intervention with TAXus and cardiac surgery; RCA, right coronary artery; GA, glycated albumin; FBG, fasting blood glucose;

HbA1c, glycosylated hemoglobin A1c.

Supplementary Table 2. The categories of all risk factors involved in studies

Categories	Risk factors involved
**Patient factors**	09, 10, 11, 12, 13, 22, 23, 25, 30, 31, 32, 33, 38, 39, 42, 45, 46, 47, 48, 49, 50, 51, 53, 54, 55, 56, 57, 60, 61, 62, 63, 64, 65, 66, 70, 71, 72
**Lesion factors**	03, 14, 15, 24, 26, 35, 37, 40, 41, 43, 58, 59, 67, 73
**Stent factors**	01, 02, 04, 06, 20, 69
**Procedural factors**	05, 07, 08, 16, 17, 18, 19, 21, 27, 28, 29, 34, 36, 44, 52, 68,

The numbers and abbreviations are represented as in Table S1.

Supplementary Table 3. Newcastle-Ottawa quality assessment scale for 6 case-control studies

	Is the case definition adequate?	Representativeness of the cases	Selection of Controls	Definition of Controls	Comparability of cases and controls on the basis of the design or analysis	Ascertainment of exposure	Same method of ascertainment for cases and controls	Non-Response rate	Total stars
Roy P, 2007	(a)☆	(a)☆	(b)	(a)☆	(a)☆	(a) (b)☆☆	(a)☆	(b)	7
Kitahara H, 2009	(a)☆	(a)☆	(b)	(a)☆	(a)☆	(a)☆	(a)☆	(b)	6
Ino Y, 2011	(a)☆	(a)☆	(b)	(a)☆	(a)☆	(a) (b)☆☆	(a)☆	(b)	7
Kim YG，2013	(a)☆	(a)☆	(b)	(a)☆	(a)☆	(a)☆	(a)☆	(b)	6
XU X, 2019	(a)☆	(a)☆	(b)	(a)☆	(a)☆	(a)☆	(a)☆	(b)	6
Li M, 2022	(a)☆	(a)☆	(b)	(a)☆	(a)☆	(a)☆	(a)☆	(b)	6

Supplementary Table 4. Newcastle-Ottawa quality assessment scale for 11 cohort studies

Studies	Representativ-eness of the exposed cohort	Selection of the nonexposed cohort	Ascertainment of exposure	Demonstration that outcome of interest was not present at start of study	Comparability of cohorts on the basis of the design or analysis	Assessment of outcome	Was follow-up long enough for outcomes to occur	Adequacy of follow up of cohorts	Total stars
Hong MK, 2006	(a)☆	(a)☆	(a)☆	(a)☆	(a)☆	(b)☆	(a)☆	(b)☆	8
Kastrati A, 2006	(a)☆	(a)☆	(a)☆	(a)☆	(a)☆	(b)☆	(a)☆	(b)☆	8
Park D-W, 2007	(a)☆	(a)☆	(a)☆	(a)☆	(a) (b)☆☆	(b)☆	(a)☆	(c)	8
Cassese S, 2014	(a)☆	(a)☆	(a)☆	(b)	(a)☆	(b)☆	(a)☆	(c)	6
Park SH, 2015	(a)☆	(a)☆	(a)☆	(a)☆	(a)☆	(b)☆	(a)☆	(c)	7
Zhao L-P, 2015	(a)☆	(a)☆	(a)☆	(a)☆	(a)☆	(b)☆	(a)☆	(c)	7
Gabbasov Z, 2018	(a)☆	(a)☆	(a)☆	(b)	(a) (b)☆☆	(b)☆	(a)☆	(d)	7
Gai M-T, 2021	(a)☆	(a)☆	(a)☆	(b)	(a)☆	(b)☆	(a)☆	(d)	6
Gupta PK, 2021	(a)☆	(a)☆	(a)☆	(b)	(a)☆	(b)☆	(a)☆	(d)	6
Zhu Y, 2021	(a)☆	(a)☆	(a)☆	(b)	(a)☆	(b)☆	(a)☆	(d)	6
Lin XL, 2022	(a)☆	(a)☆	(a)☆	(b)	(a)☆	(b)☆	(a)☆	(d)	6

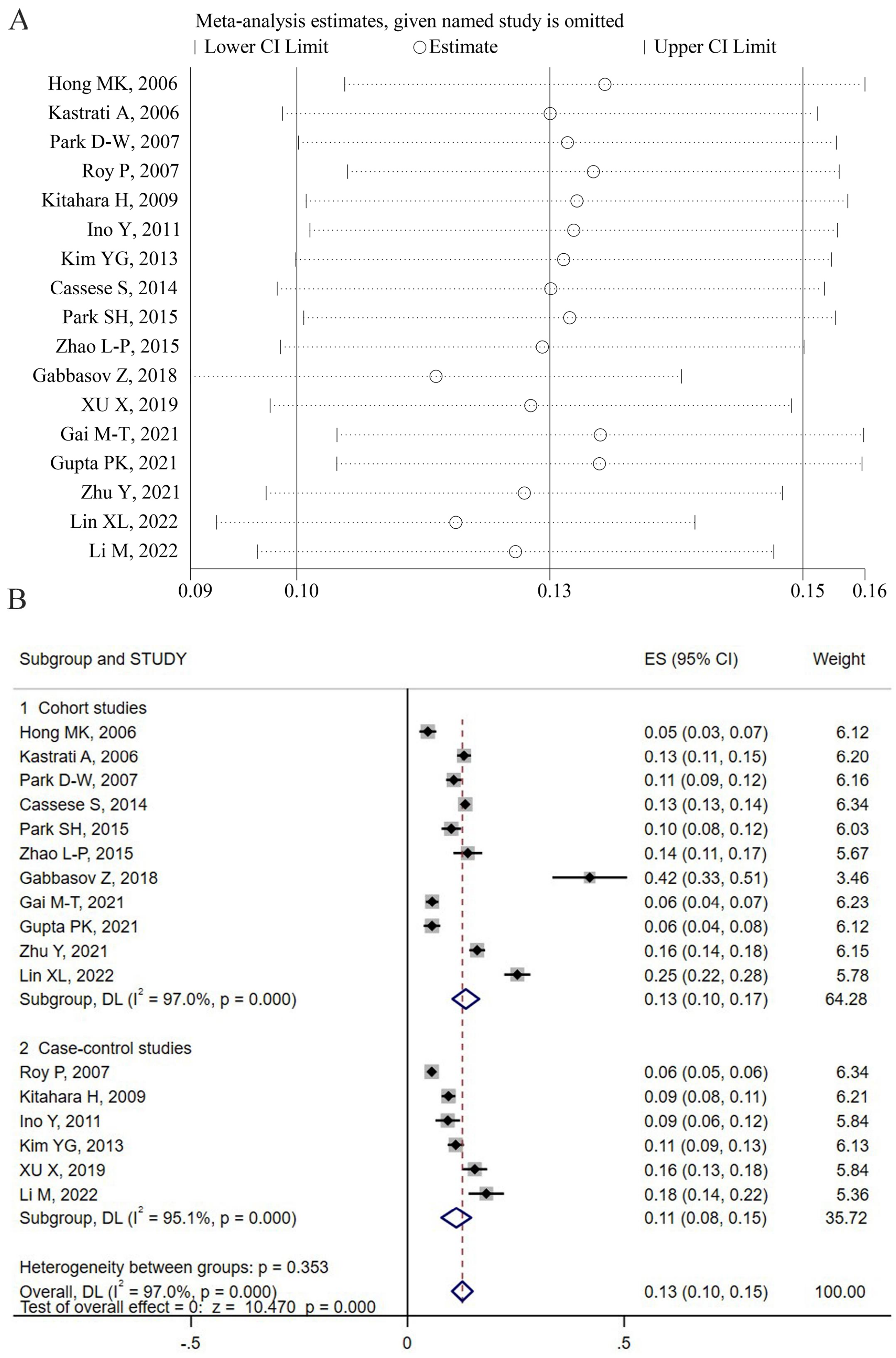

Supplementary Fig. 1. Sensitivity analysis and subgroup analysis about the incidence of DES-ISR. A. Sensitivity analysis was performed by one-by-one exclusion method; B. Forest plot of the incidence of DES-ISR and subgroup analysis according to study design.

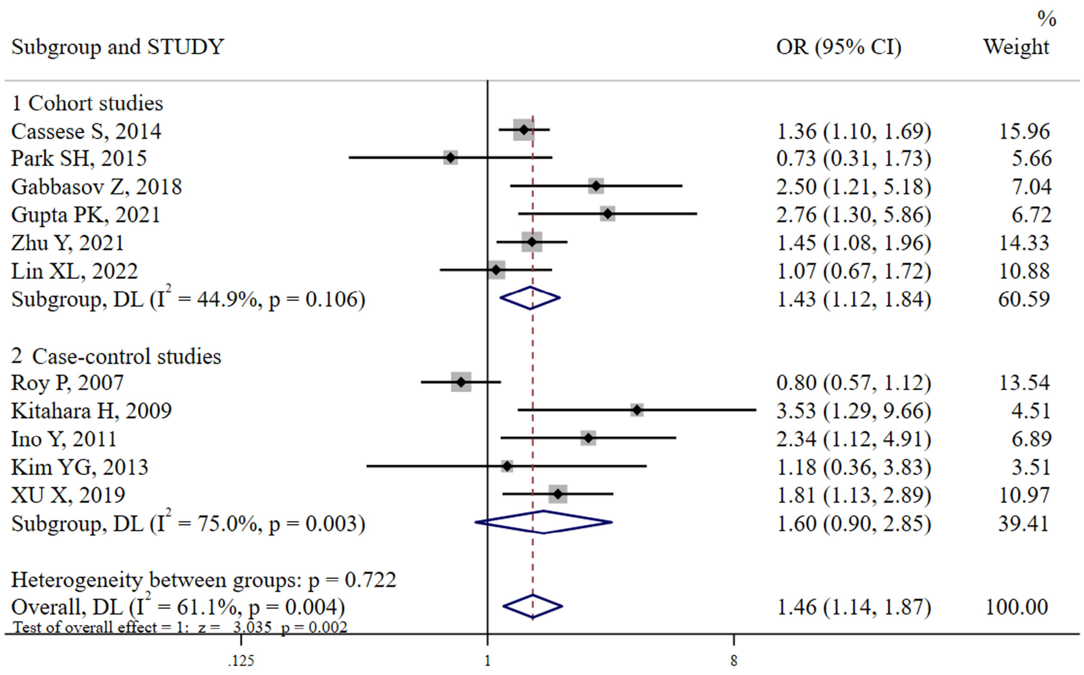

Supplementary Fig. 2. Forest plot of DM as a risk factor for DES-ISR.

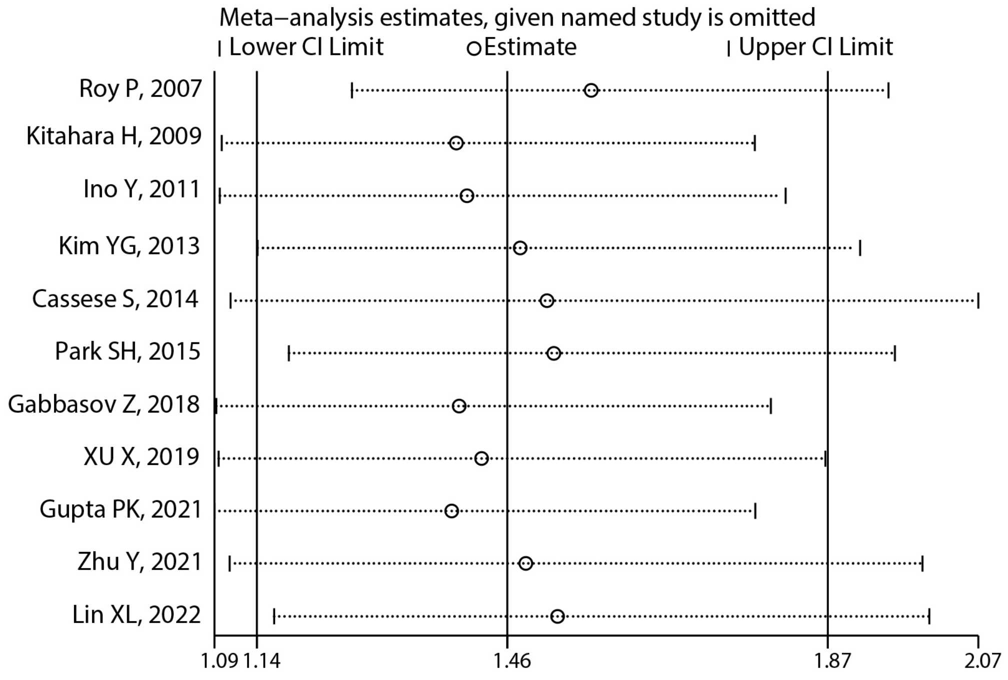

Supplementary Fig. 3. Sensitivity analysis of DM as a risk factor for DES-ISR.

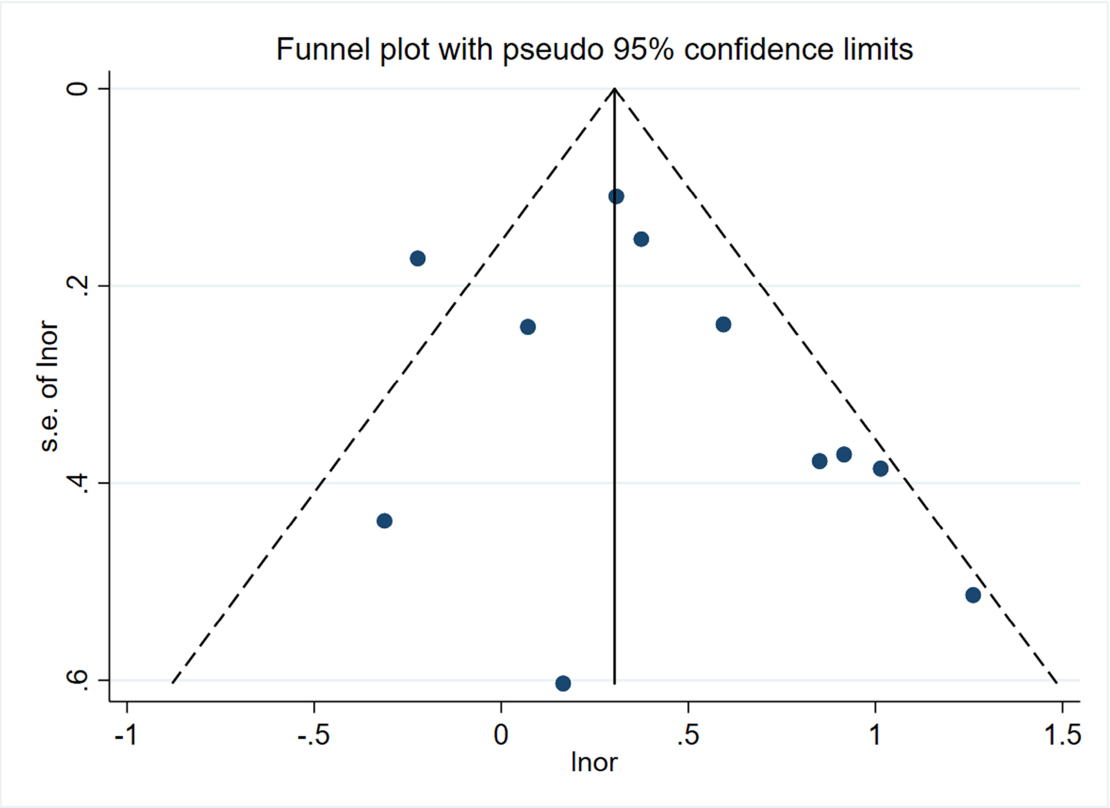

Supplementary Fig. 4. Funnel plot of the studies that record DM as a risk factor for DES-ISR.

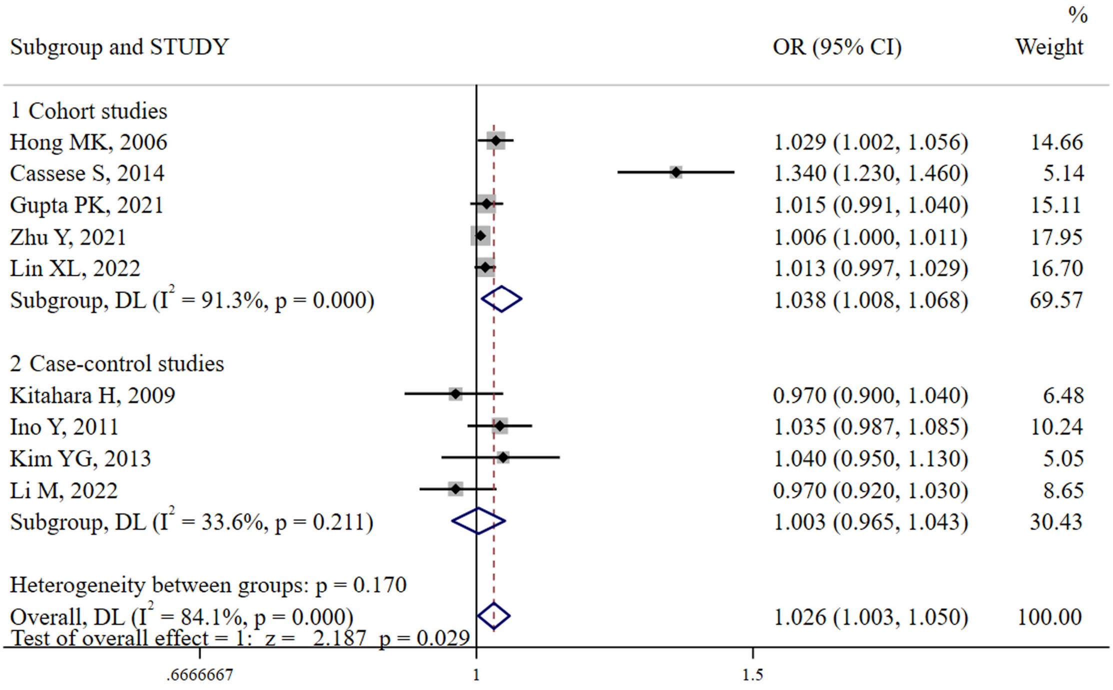

Supplementary Fig. 5. Forest plot of stent length (mm) as a risk factor for DES-ISR.

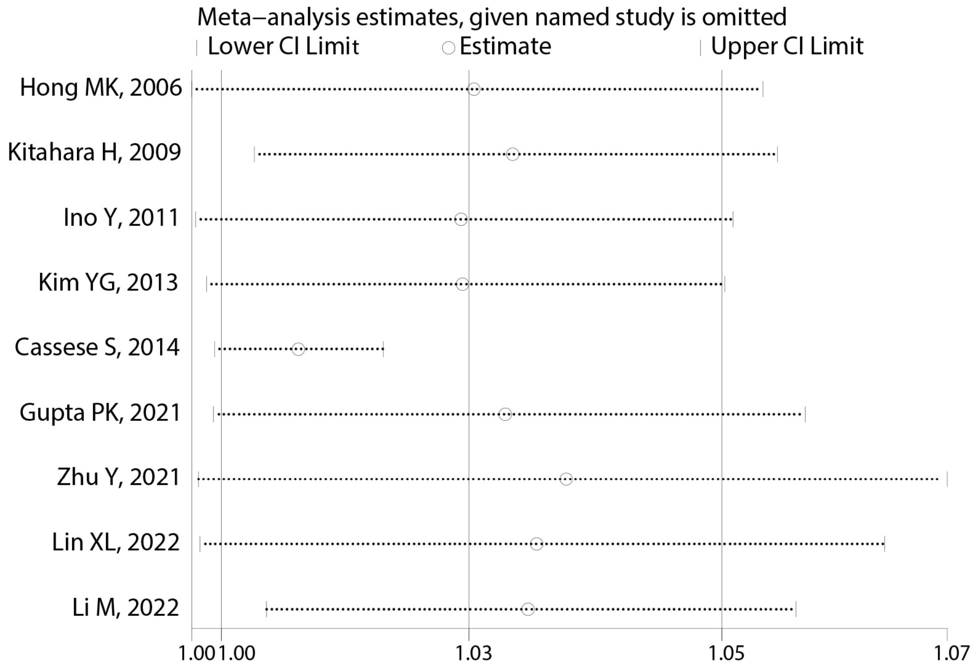

Supplementary Fig. 6. **Sensitivity analysis of stent length as a risk factor for DES-ISR.**

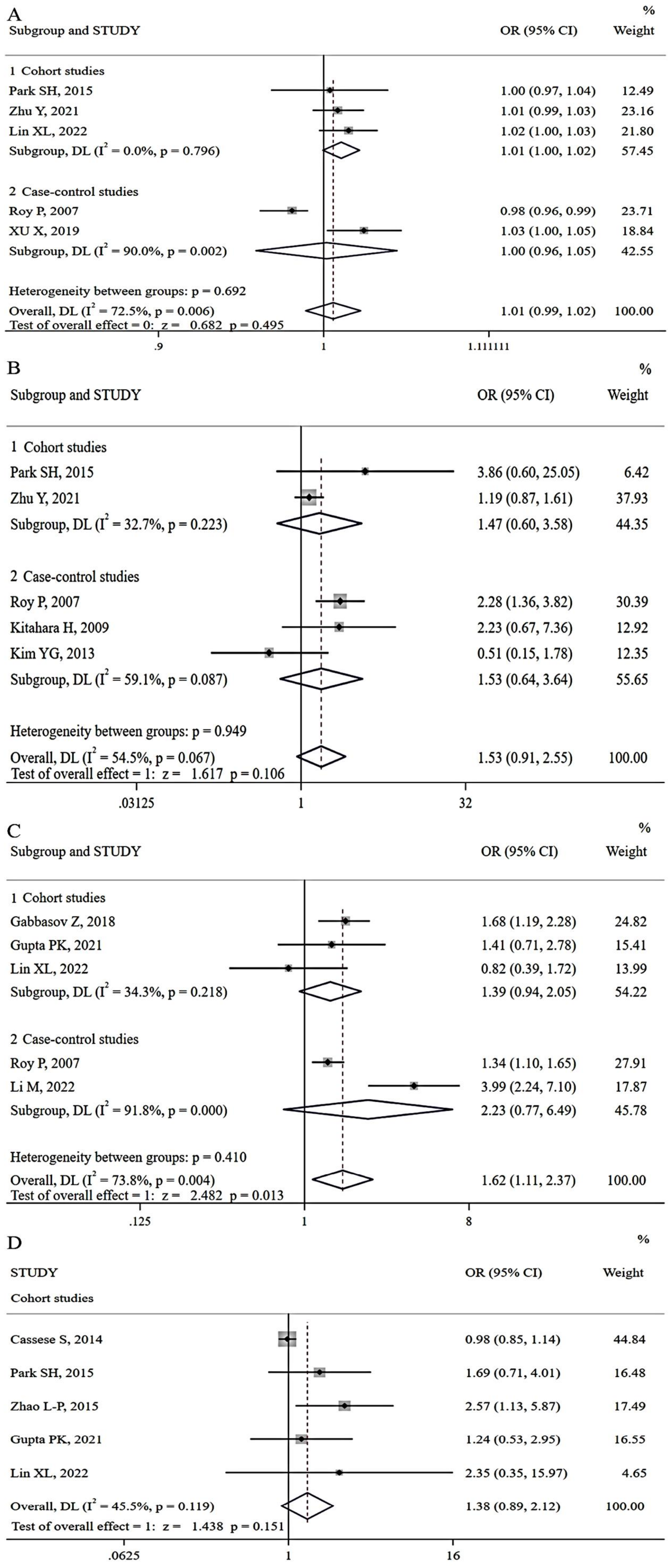
A
%
Subgroup and STUDY
OR (95% CI)
Weight
1 Cohort studies
Park SH, 2015
1.00 (0.97, 1.04)
12.49
Zhu Y, 2021
1.01 (0.99, 1.03)
23.16
Lin XL, 2022
1.02 (1.00, 1.03)
21.80
Subgroup, DL (I² = 0.0%, p = 0.796)
1.01 (1.00, 1.02)
57.45
2 Case-control studies
Roy P, 2007
0.98 (0.96, 0.99)
23.71
XU X, 2019
1.03 (1.00, 1.05)
18.84
Subgroup, DL (I² = 90.0%, p = 0.002)
1.00 (0.96, 1.05)
42.55
Heterogeneity between groups: p = 0.692
Overall, DL (I² = 72.5%, p = 0.006)
1.01 (0.99, 1.02)
100.00
Test of overall effect = 0: z = 0.682 p = 0.495
.9
1
1.111111
B
%
Subgroup and STUDY
OR (95% CI)
Weight
1 Cohort studies
Park SH, 2015
3.86 (0.60, 25.05)
6.42
Zhu Y, 2021
1.19 (0.87, 1.61)
37.93
Subgroup, DL (I² = 32.7%, p = 0.223)
1.47 (0.60, 3.58)
44.35
2 Case-control studies
Roy P, 2007
2.28 (1.36, 3.82)
30.39
Kitahara H, 2009
2.23 (0.67, 7.36)
12.92
Kim YG, 2013
0.51 (0.15, 1.78)
12.35
Subgroup, DL (I² = 59.1%, p = 0.087)
1.53 (0.64, 3.64)
55.65
Heterogeneity between groups: p = 0.949
Overall, DL (I² = 54.5%, p = 0.067)
1.53 (0.91, 2.55)
100.00
Test of overall effect = 1: z = 1.617 p = 0.106
.03125
1
32
C
%
Subgroup and STUDY
OR (95% CI)
Weight
1 Cohort studies
Gabbasov Z, 2018
1.68 (1.19, 2.28)
24.82
Gupta PK, 2021
1.41 (0.71, 2.78)
15.41
Lin XL, 2022
0.82 (0.39, 1.72)
13.99
Subgroup, DL (I² = 34.3%, p = 0.218)
1.39 (0.94, 2.05)
54.22
2 Case-control studies
Roy P, 2007
1.34 (1.10, 1.65)
27.91
Li M, 2022
3.99 (2.24, 7.10)
17.87
Subgroup, DL (I² = 91.8%, p = 0.000)
2.23 (0.77, 6.49)
45.78
Heterogeneity between groups: p = 0.410
Overall, DL (I² = 73.8%, p = 0.004)
1.62 (1.11, 2.37)
100.00
Test of overall effect = 1: z = 2.482 p = 0.013
.125
1
8
D
%
STUDY
OR (95% CI)
Weight
Cohort studies
Cassese S, 2014
0.98 (0.85, 1.14)
44.84
Park SH, 2015
1.69 (0.71, 4.01)
16.48
Zhao L-P, 2015
2.57 (1.13, 5.87)
17.49
Gupta PK, 2021
1.24 (0.53, 2.95)
16.55
Lin XL, 2022
2.35 (0.35, 15.97)
4.65
Overall, DL (I² = 45.5%, p = 0.119)
1.38 (0.89, 2.12)
100.00
Test of overall effect = 1: z = 1.438 p = 0.151
.0625
1
16

Supplementary Fig. 7. **Forest plot of the risk factors reported in 5 studies. A. Age; B. Hypertension; C. Number of stents; D. Multivessel disease.**

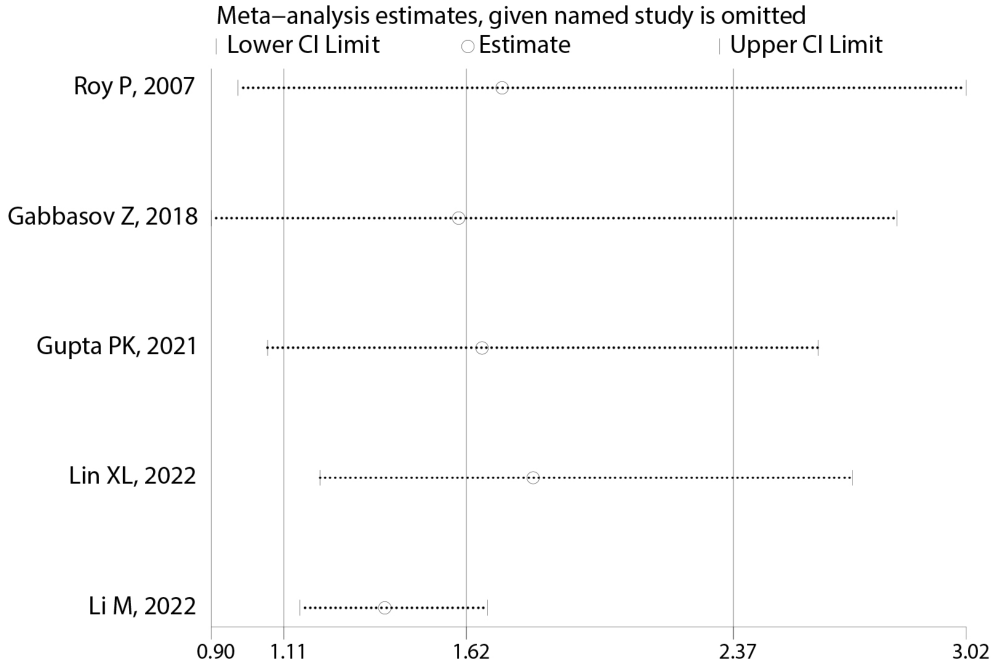

Supplementary Fig. 8 Sensitivity analysis of the number of stents as a risk factor for DES-ISR.

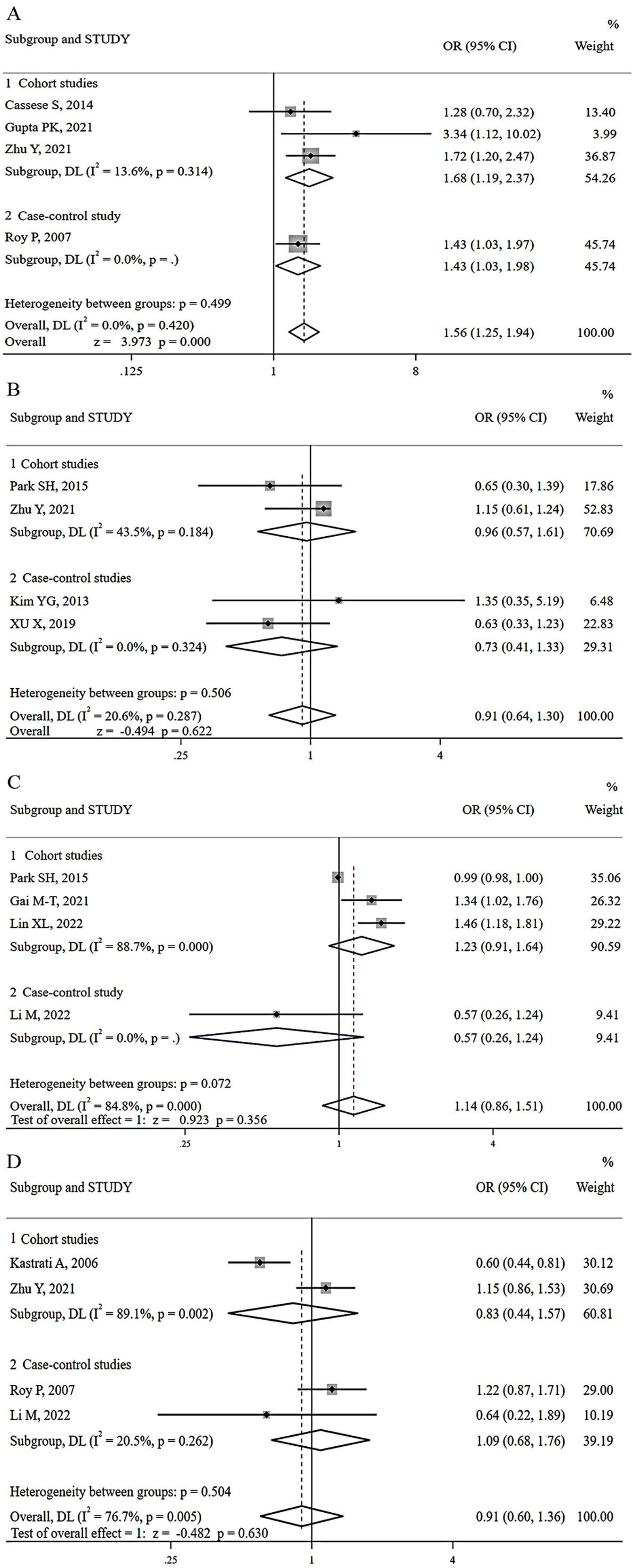
A
Subgroup and STUDY
OR (95% CI)
% Weight
1 Cohort studies
Cassese S, 2014
1.28 (0.70, 2.32)
13.40
Gupta PK, 2021
3.34 (1.12, 10.02)
3.99
Zhu Y, 2021
1.72 (1.20, 2.47)
36.87
Subgroup, DL (I2 = 13.6%, p = 0.314)
1.68 (1.19, 2.37)
54.26
2 Case-control study
Roy P, 2007
1.43 (1.03, 1.97)
45.74
Subgroup, DL (I2 = 0.0%, p = .)
1.43 (1.03, 1.98)
45.74
Heterogeneity between groups: p = 0.499
Overall, DL (I2 = 0.0%, p = 0.420)
1.56 (1.25, 1.94)
100.00
Overall z = 3.973 p = 0.000
.125
1
8
B
Subgroup and STUDY
OR (95% CI)
% Weight
1 Cohort studies
Park SH, 2015
0.65 (0.30, 1.39)
17.86
Zhu Y, 2021
1.15 (0.61, 1.24)
52.83
Subgroup, DL (I2 = 43.5%, p = 0.184)
0.96 (0.57, 1.61)
70.69
2 Case-control studies
Kim YG, 2013
1.35 (0.35, 5.19)
6.48
XU X, 2019
0.63 (0.33, 1.23)
22.83
Subgroup, DL (I2 = 0.0%, p = 0.324)
0.73 (0.41, 1.33)
29.31
Heterogeneity between groups: p = 0.506
Overall, DL (I2 = 20.6%, p = 0.287)
0.91 (0.64, 1.30)
100.00
Overall z = -0.494 p = 0.622
.25
1
4
C
Subgroup and STUDY
OR (95% CI)
% Weight
1 Cohort studies
Park SH, 2015
0.99 (0.98, 1.00)
35.06
Gai M-T, 2021
1.34 (1.02, 1.76)
26.32
Lin XL, 2022
1.46 (1.18, 1.81)
29.22
Subgroup, DL (I2 = 88.7%, p = 0.000)
1.23 (0.91, 1.64)
90.59
2 Case-control study
Li M, 2022
0.57 (0.26, 1.24)
9.41
Subgroup, DL (I2 = 0.0%, p = .)
0.57 (0.26, 1.24)
9.41
Heterogeneity between groups: p = 0.072
Overall, DL (I2 = 84.8%, p = 0.000)
1.14 (0.86, 1.51)
100.00
Test of overall effect = 1: z = 0.923 p = 0.356
.25
1
4
D
Subgroup and STUDY
OR (95% CI)
% Weight
1 Cohort studies
Kastrati A, 2006
0.60 (0.44, 0.81)
30.12
Zhu Y, 2021
1.15 (0.86, 1.53)
30.69
Subgroup, DL (I2 = 89.1%, p = 0.002)
0.83 (0.44, 1.57)
60.81
2 Case-control studies
Roy P, 2007
1.22 (0.87, 1.71)
29.00
Li M, 2022
0.64 (0.22, 1.89)
10.19
Subgroup, DL (I2 = 20.5%, p = 0.262)
1.09 (0.68, 1.76)
39.19
Heterogeneity between groups: p = 0.504
Overall, DL (I2 = 76.7%, p = 0.005)
0.91 (0.60, 1.36)
100.00
Test of overall effect = 1: z = -0.482 p = 0.630
.25
1
4

Supplementary Fig. 9. **Forest plot of the risk factors reported in 4 studies. A. LAD; B. Male; C. LDL-C; D. Sirolimus-eluting stents.**

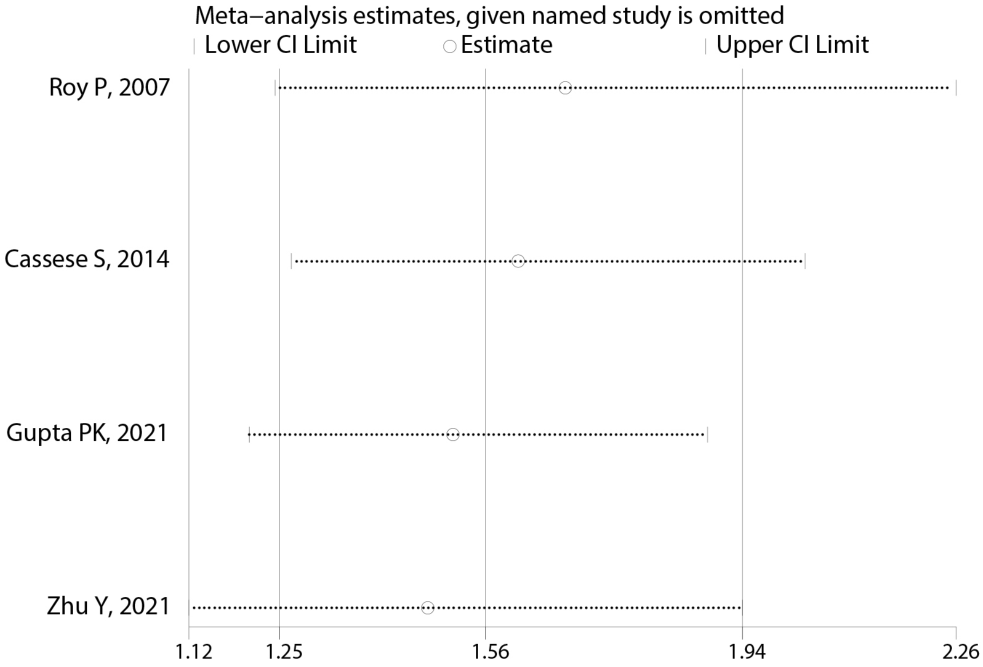

Supplementary Fig. 10. **Sensitivity analysis of LAD as a risk factor for DES-ISR.**

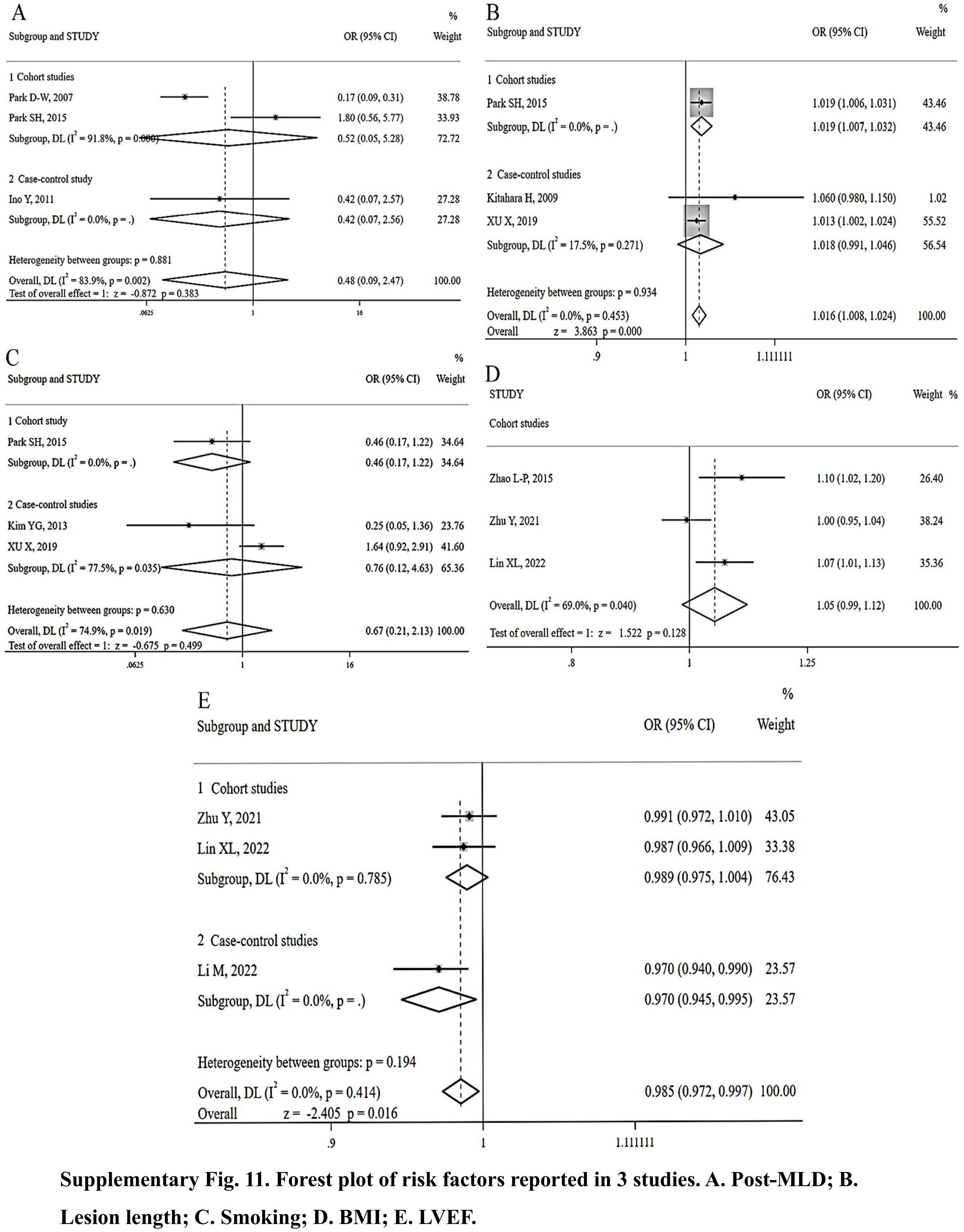

Supplementary Fig. 11. Forest plot of risk factors reported in 3 studies. A. Post-MLD; B. Lesion length; C. Smoking; D. BMI; E. LVEF.

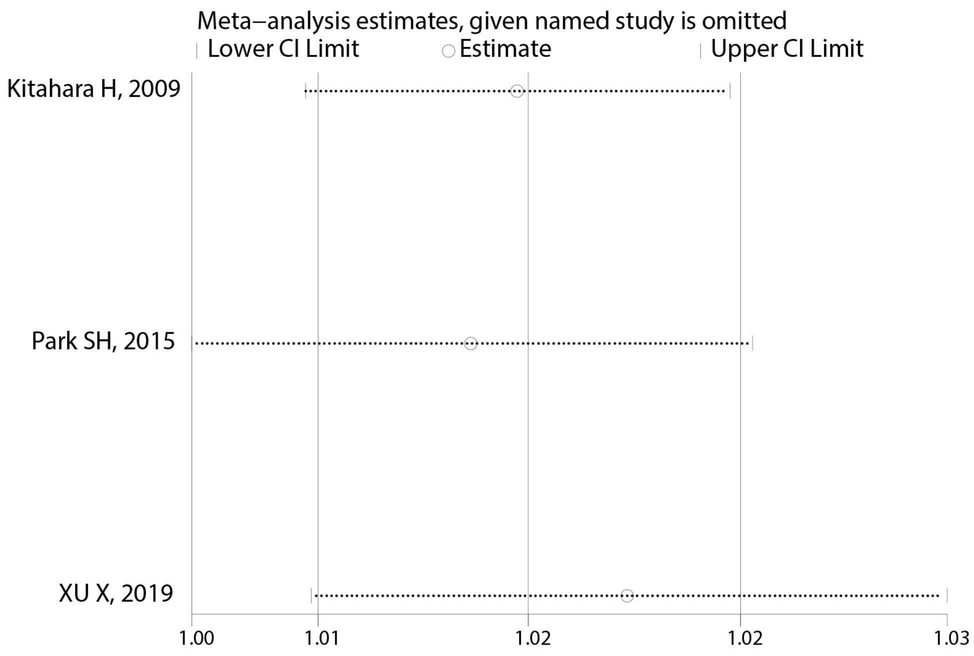

Supplementary Fig. 12. **Sensitivity analysis of lesion length as a risk factor for DES-ISR.**

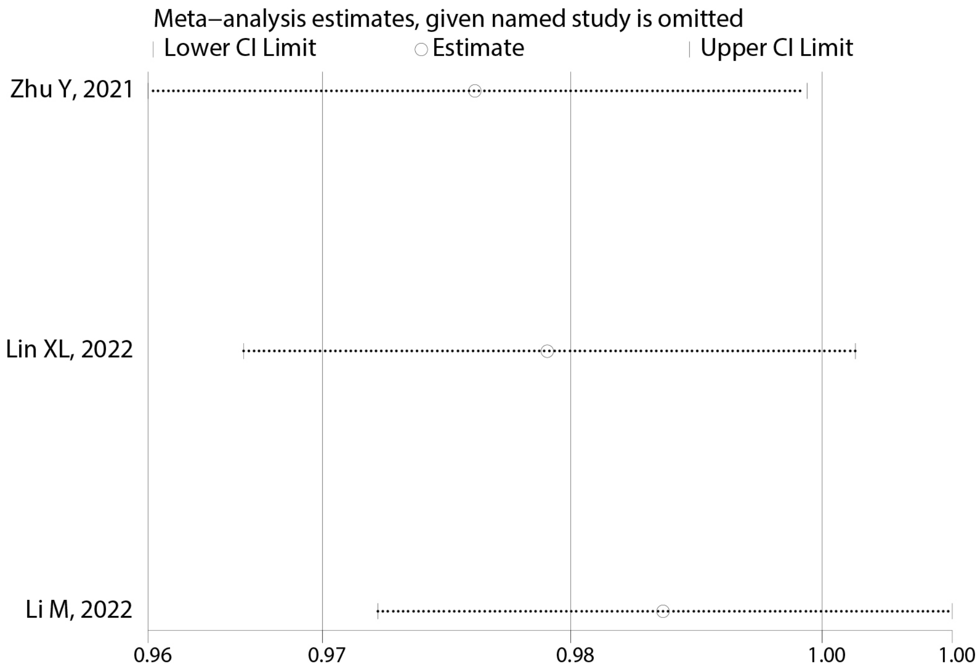

Supplementary Fig. 13. **Sensitivity analysis of LVEF as a risk factor for DES-ISR.**

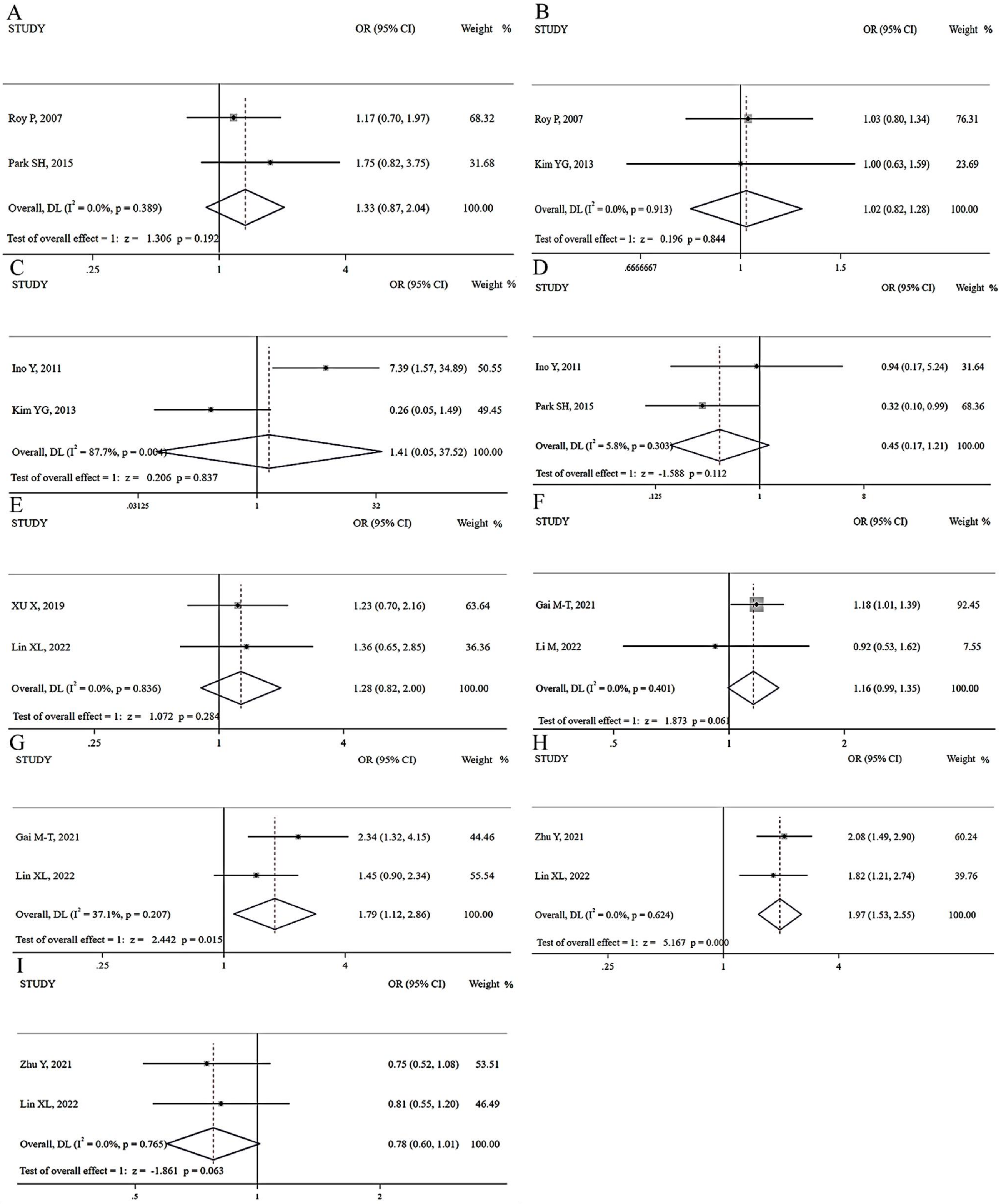

Supplementary Fig. 14. Forest plot of risk factors reported in 2 studies. A. Dyslipidemia; B. Stent diameter; C. Aorta ostium stenting; D. RVD; E. Multiple stents; F. TC; G. Medical history of MI; H. Previous PCI; I. Minimal stent diameter.

MOOSE (Meta-analyses Of Observational Studies in Epidemiology) Checklist

A reporting checklist for Authors, Editors, and Reviewers of Meta-analyses of Observational Studies. You must report the page number in your manuscript where you consider each of the items listed in this checklist. If you have not included this information, either revise your manuscript accordingly before submitting or note N/A.

Reporting Criteria	Reported (Yes/No)	Reported on Page No.
**Reporting of Background**		
Problem definition		
Hypothesis statement		
Description of Study Outcome(s)		
Type of exposure or intervention used		
Type of study design used		
Study population		
**Reporting of Search Strategy**		
Qualifications of searchers (eg, librarians and investigators)		
Search strategy, including time period included in the synthesis and keywords		
Effort to include all available studies, including contact with authors		
Databases and registries searched		
Search software used, name and version, including special features used (eg, explosion)		
Use of hand searching (eg, reference lists of obtained articles)		
List of citations located and those excluded, including justification		
Method for addressing articles published in languages other than English		
Method of handling abstracts and unpublished studies		
Description of any contact with authors		
**Reporting of Methods**		
Description of relevance or appropriateness of studies assembled for assessing the hypothesis to be tested		
Rationale for the selection and coding of data (eg, sound clinical principles or convenience)		
Documentation of how data were classified and coded (eg, multiple raters, blinding, and interrater reliability)		
Assessment of confounding (eg, comparability of cases and controls in studies where appropriate		

Reporting Criteria	Reported (Yes/No)	Reported on Page No.
Assessment of study quality, including blinding of quality assessors; stratification or regression on possible predictors of study results		
Assessment of heterogeneity		
Description of statistical methods (eg, complete description of fixed or random effects models, justification of whether the chosen models account for predictors of study results, dose-response models, or cumulative meta-analysis) in sufficient detail to be replicated		
Provision of appropriate tables and graphics		
**Reporting of Results**		
Table giving descriptive information for each study included		
Results of sensitivity testing (eg, subgroup analysis)		
Indication of statistical uncertainty of findings		
**Reporting of Discussion**		
Quantitative assessment of bias (eg, publication bias)		
Justification for exclusion (eg, exclusion of non–English-language citations)		
Assessment of quality of included studies		
**Reporting of Conclusions**		
Consideration of alternative explanations for observed results		
Generalization of the conclusions (ie, appropriate for the data presented and within the domain of the literature review)		
Guidelines for future research		
Disclosure of funding source		

Once you have completed this checklist, please save a copy and upload it as part of your submission. DO NOT include this checklist as part of the main manuscript document. It must be uploaded as a separate file.

NEWCASTLE - OTTAWA QUALITY ASSESSMENT SCALE
CASE CONTROL STUDIES

Note: A study can be awarded a maximum of one star for each numbered item within the Selection and Exposure categories. A maximum of two stars can be given for Comparability.

Selection

1) Is the case definition adequate?
   a) yes, with independent validation ✵
   b) yes, eg record linkage or based on self reports
   c) no description

2) Representativeness of the cases
   a) consecutive or obviously representative series of cases ✵
   b) potential for selection biases or not stated

3) Selection of Controls
   a) community controls ✵
   b) hospital controls
   c) no description

4) Definition of Controls
   a) no history of disease (endpoint) ✵
   b) no description of source

Comparability

1) Comparability of cases and controls on the basis of the design or analysis
   a) study controls for ________________ (Select the most important factor.) ✵
   b) study controls for any additional factor ✵ (This criteria could be modified to indicate specific control for a second important factor.)

Exposure

1) Ascertainment of exposure
   a) secure record (eg surgical records) ✵
   b) structured interview where blind to case/control status ✵
   c) interview not blinded to case/control status
   d) written self report or medical record only
   e) no description

2) Same method of ascertainment for cases and controls
   a) yes ✵
   b) no

3) Non-Response rate
   a) same rate for both groups ✵
   b) non respondents described
   c) rate different and no designation

NEWCASTLE - OTTAWA QUALITY ASSESSMENT SCALE
COHORT STUDIES

Note: A study can be awarded a maximum of one star for each numbered item within the Selection and Outcome categories. A maximum of two stars can be given for Comparability

Selection

1) Representativeness of the exposed cohort
   a) truly representative of the average _______________ (describe) in the community ❋
   b) somewhat representative of the average ______________ in the community ❋
   c) selected group of users eg nurses, volunteers
   d) no description of the derivation of the cohort

2) Selection of the non exposed cohort
   a) drawn from the same community as the exposed cohort ❋
   b) drawn from a different source
   c) no description of the derivation of the non exposed cohort

3) Ascertainment of exposure
   a) secure record (eg surgical records) ❋
   b) structured interview ❋
   c) written self report
   d) no description

4) Demonstration that outcome of interest was not present at start of study
   a) yes ❋
   b) no

Comparability

1) Comparability of cohorts on the basis of the design or analysis
   a) study controls for _____________ (select the most important factor) ❋
   b) study controls for any additional factor ❋ (This criteria could be modified to indicate specific control for a second important factor.)

Outcome

1) Assessment of outcome
   a) independent blind assessment ❋
   b) record linkage ❋
   c) self report
   d) no description

2) Was follow-up long enough for outcomes to occur
   a) yes (select an adequate follow up period for outcome of interest) ❋
   b) no

3) Adequacy of follow up of cohorts
   a) complete follow up - all subjects accounted for ❋
   b) subjects lost to follow up unlikely to introduce bias - small number lost - > ____ % (select an adequate %) follow up, or description provided of those lost) ❋
   c) follow up rate < ____% (select an adequate %) and no description of those lost
   d) no statement

NEWCASTLE - OTTAWA QUALITY ASSESSMENT SCALE
(adapted for cross-sectional studies)

Selection: (Maximum 3 stars)

1) Representativeness of the sample:
   a) Truly representative of the average in the target population. ✸ (all subjects or random sampling)
   b) Somewhat representative of the average in the target population. ✸ (non-random sampling)
   c) Selected group of users.
   d) No description of the sampling strategy.

2) Non-respondents:
   a) Comparability between respondents and non-respondents characteristics is established, and the response rate is satisfactory. ✸
   b) The response rate is unsatisfactory, or the comparability between respondents and non-respondents is unsatisfactory.
   c) No description of the response rate or the characteristics of the responders and the non-responders.

3) Ascertainment of the exposure (risk factor):
   a) Validated measurement tool. ✸
   b) Non-validated measurement tool, but the tool is available or described.
   c) No description of the measurement tool.

Comparability: (Maximum 2 stars)

1) The subjects in different outcome groups are comparable, based on the study design or analysis. Confounding factors are controlled.
   a) The study controls for the most important factor (select one). ✸
   b) The study control for any additional factor. ✸

Outcome: (Maximum 2 stars)

1) Assessment of the outcome:
   a) Independent blind assessment. ✸
   b) Record linkage. ✸
   c) Self report.
   d) No description.

2) Statistical test:
   a) The statistical test used to analyze the data is clearly described and appropriate, and the measurement of the association is presented, including confidence intervals and the probability level (p value). ✸
   b) The statistical test is not appropriate, not described or incomplete.

This scale has been adapted from the Newcastle-Ottawa Quality Assessment Scale for cohort and case-control studies to perform a quality assessment of cross-sectional studies for the systematic review, “Exposure to second-hand smoke and the risk of tuberculosis in children and adults: systematic review and a meta-analysis of 18 observational studies”. This scale was a modified version of the NOS scale, as also used by several other studies that have felt the need to adapt the NOS scale so as to appropriately assess the quality of cross-sectional studies.

We did a comprehensive search on literature and found that a NOS score of 7 or more can be considered a “good” study (see McPheeters et al. 2012; see Appendix G page 103-104 in http://www.ncbi.nlm.nih.gov/pubmedhealth/PMH0049229/). So we used this criterion as a cut off for good quality study.

References

Wells, G. A., Shea, B., O'Connell, D., Peterson, J., Welch, V., et al. The Newcastle-Ottawa Scale (NOS) for assessing the quality of nonrandomized studies in meta-analysis. 2011. http://www.ohri.ca/programs/clinical_epidemiology/oxford.asp

McPheeters, M.L., Kripalini, S., Peterson, N.B., Idowu, R.T. et al. (2012). Quality Improvement Interventions To Address Health Disparities. Evidence Report/ technology Assessment. Rockville (MD): Agency for Healthcare Research and Quality (US). http://www.ncbi.nlm.nih.gov/pubmedhealth/PMH0049222/pdf/TOC.pdf
