# Supplementary figures and images for: Risk Factors and Incidence for In-Stent Restenosis with Drug-Eluting Stent: A Systematic Review and Meta-Analysis
Source: Rev Cardiovasc Med. 2024 Dec 24;25(12):458. doi: 10.31083/j.rcm2512458 (PMC11683691; doi:10.31083/j.rcm2512458)

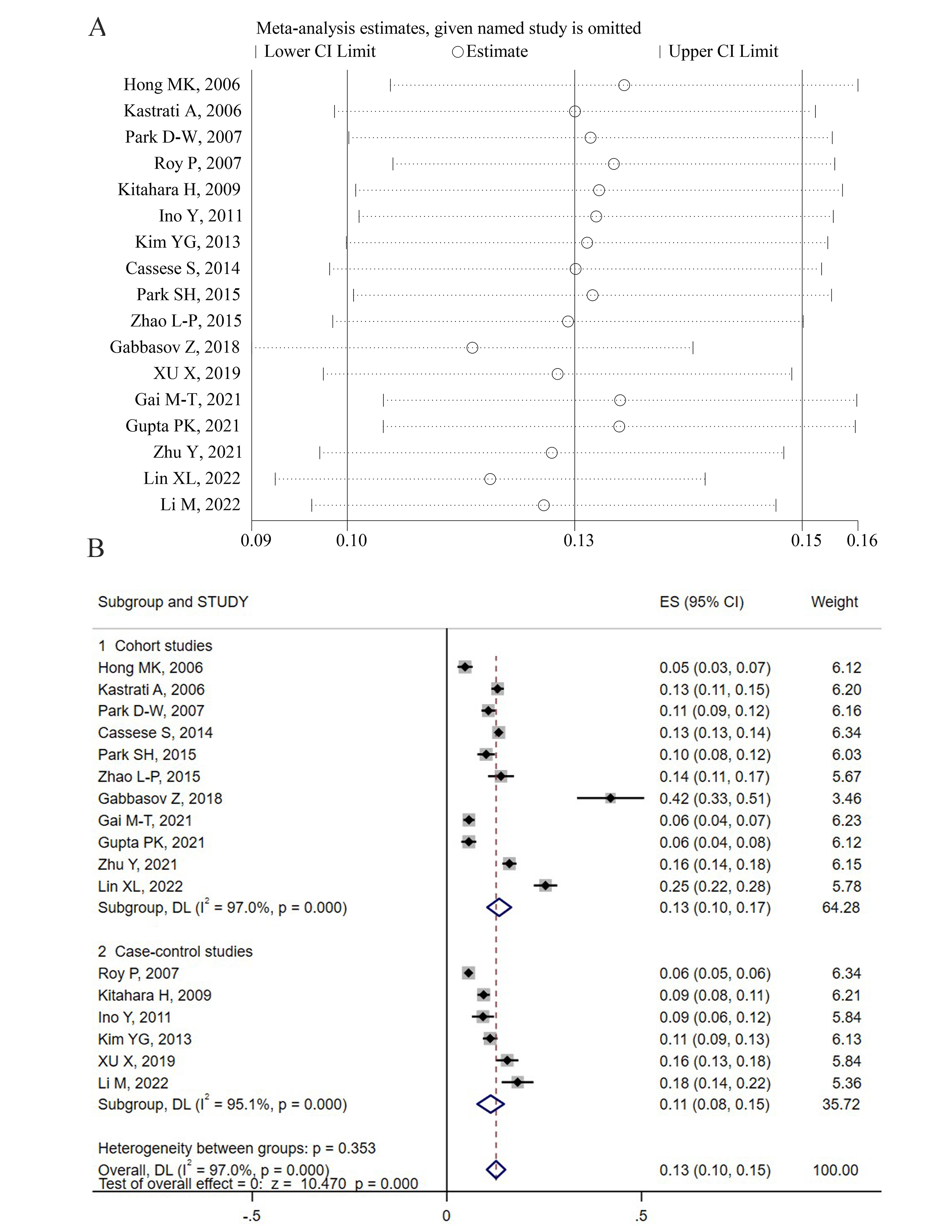

Supplement: Supplementary file 1 [file 2153-8174-25-12-458-s1.zip › Supplementary Fig. 1.jpg]

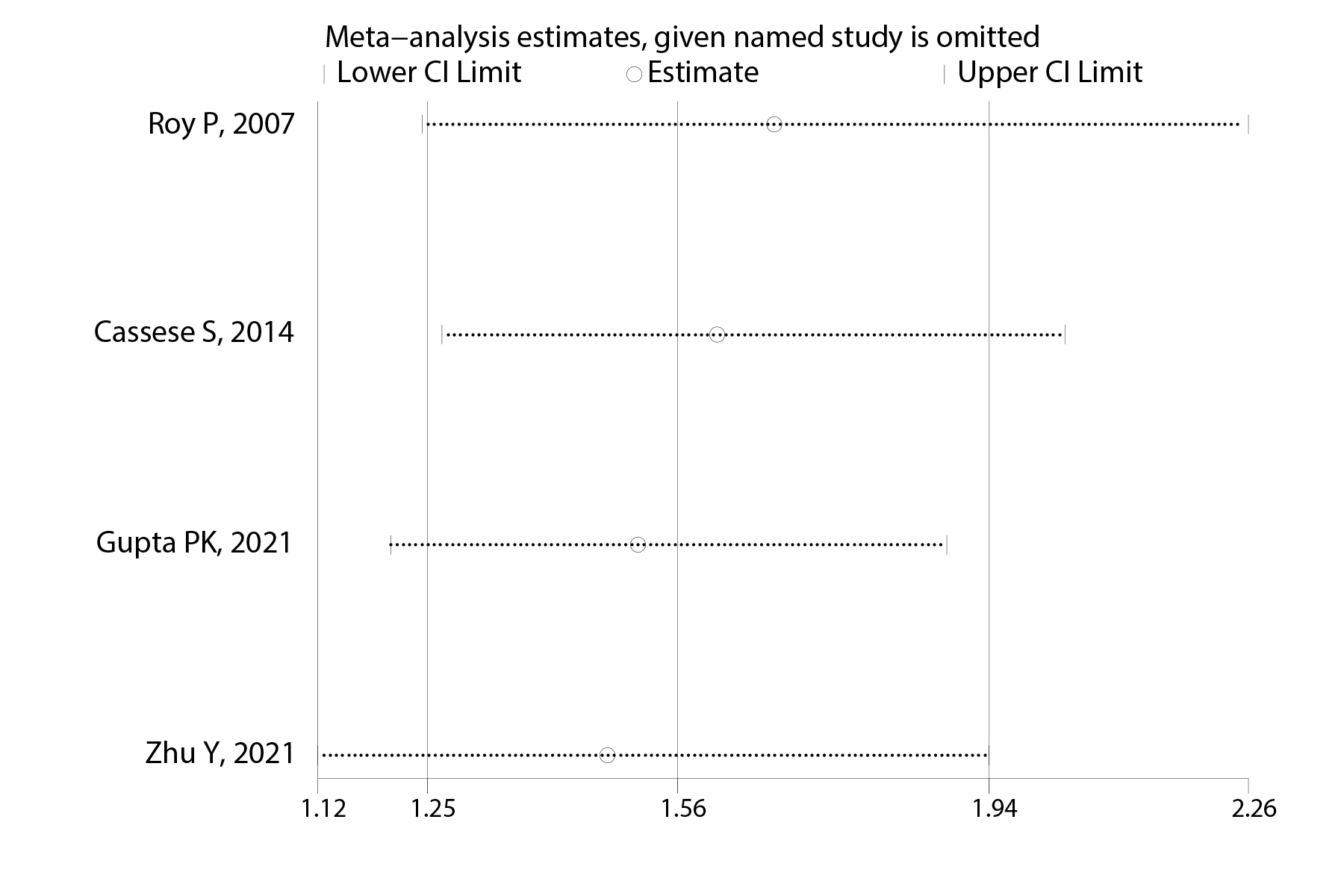

Supplement: Supplementary file 1 [file 2153-8174-25-12-458-s1.zip › Supplementary Fig. 10.jpg]

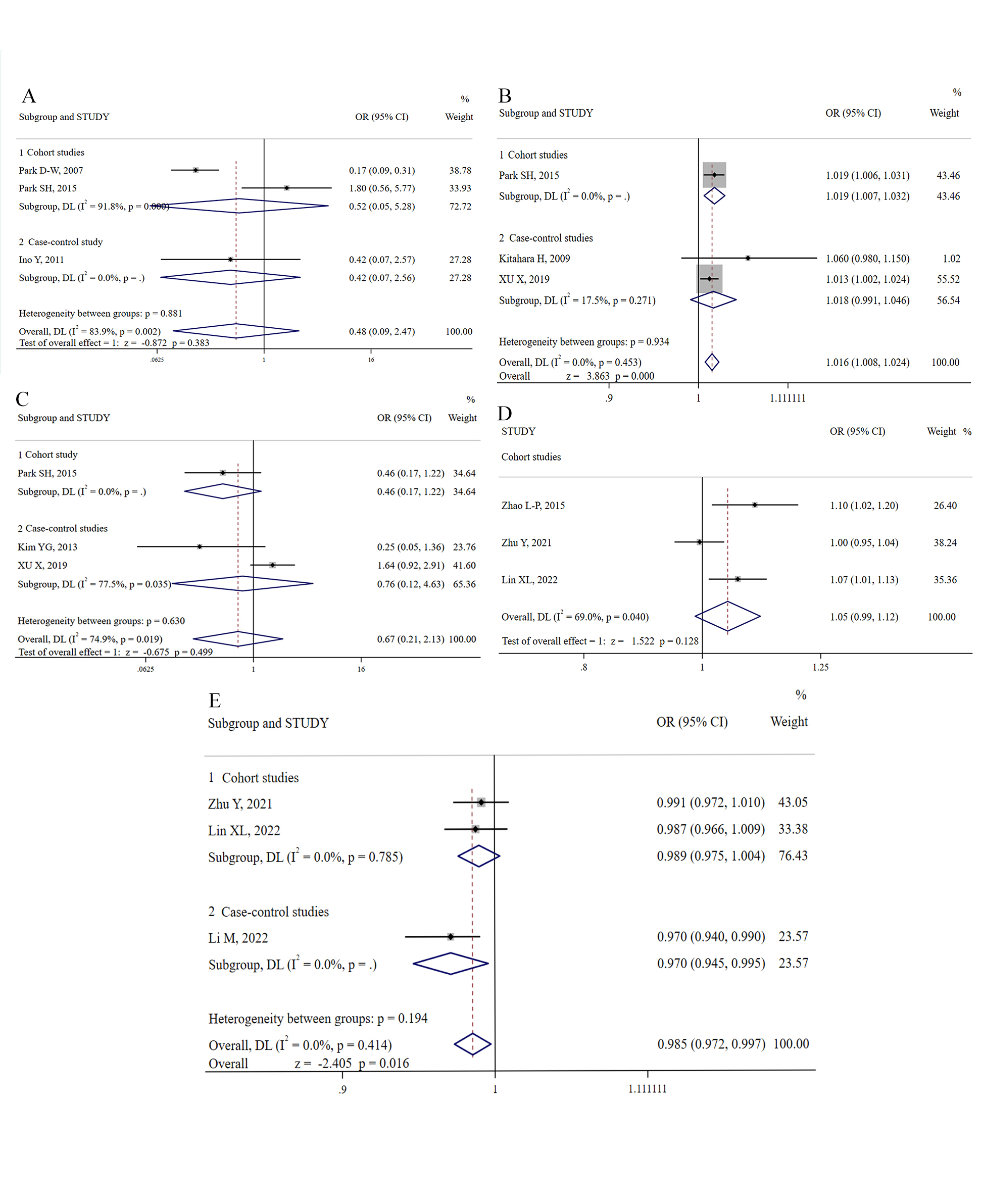

Supplement: Supplementary file 1 [file 2153-8174-25-12-458-s1.zip › Supplementary Fig. 11.jpg]

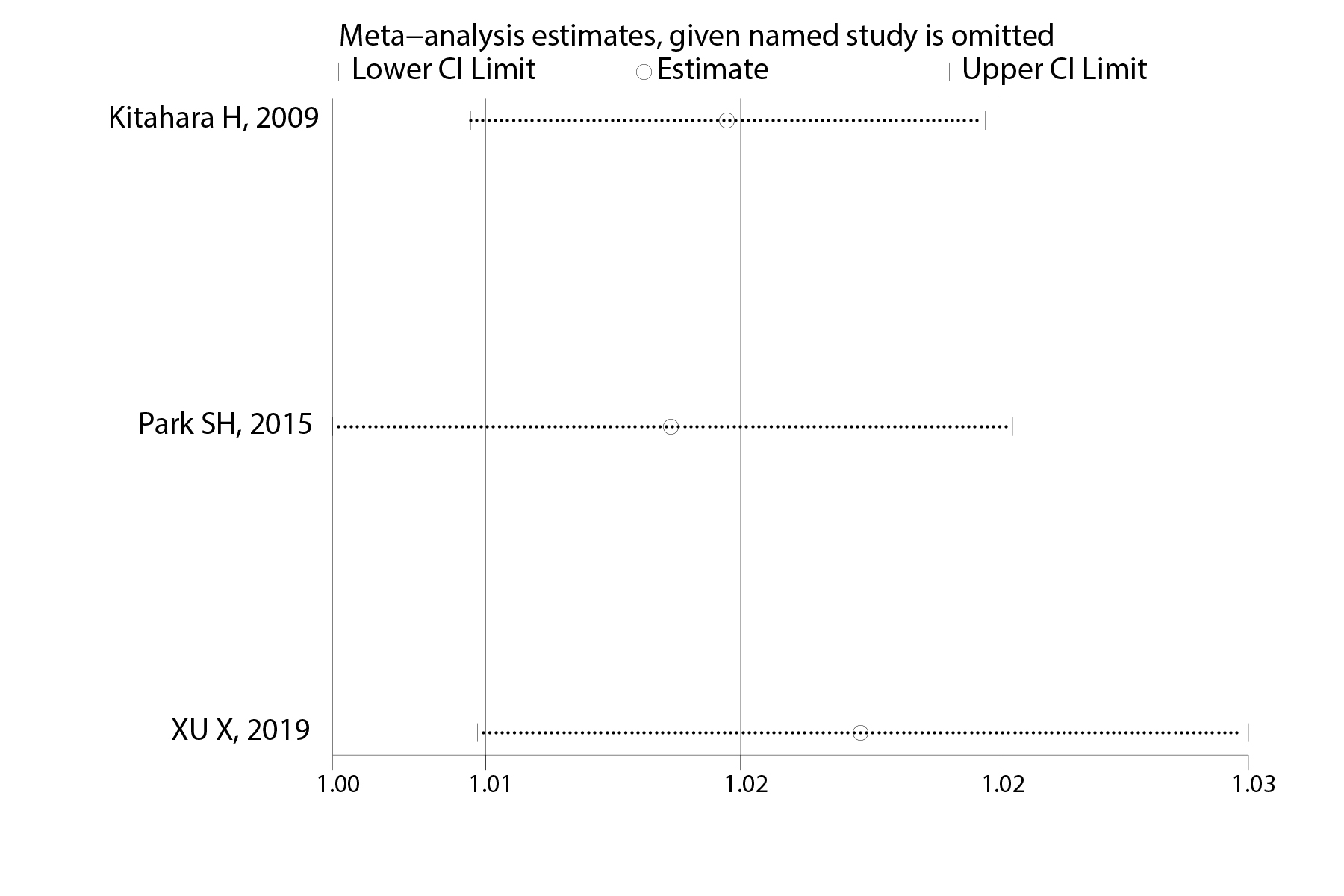

Supplement: Supplementary file 1 [file 2153-8174-25-12-458-s1.zip › Supplementary Fig. 12.jpg]

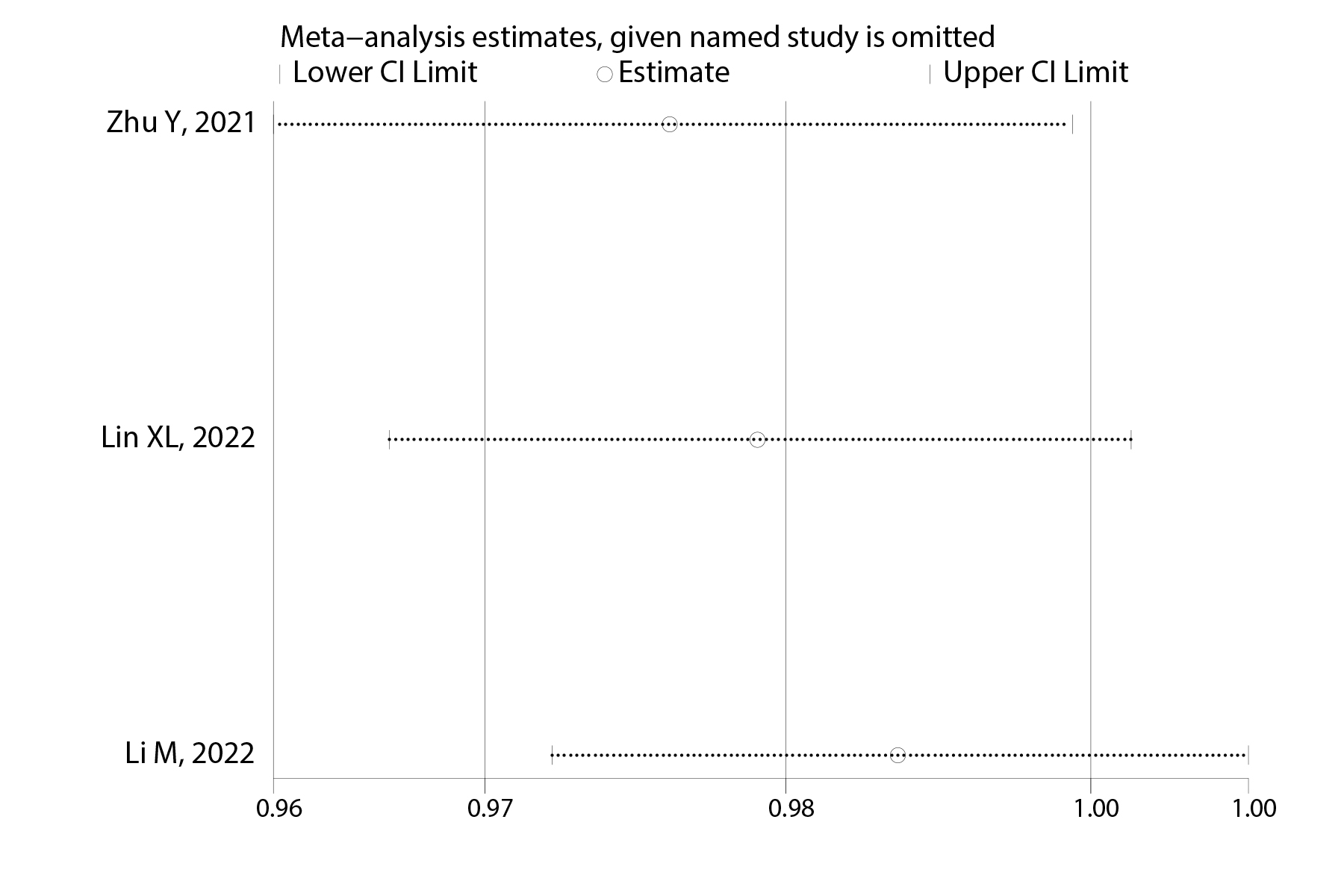

Supplement: Supplementary file 1 [file 2153-8174-25-12-458-s1.zip › Supplementary Fig. 13.jpg]

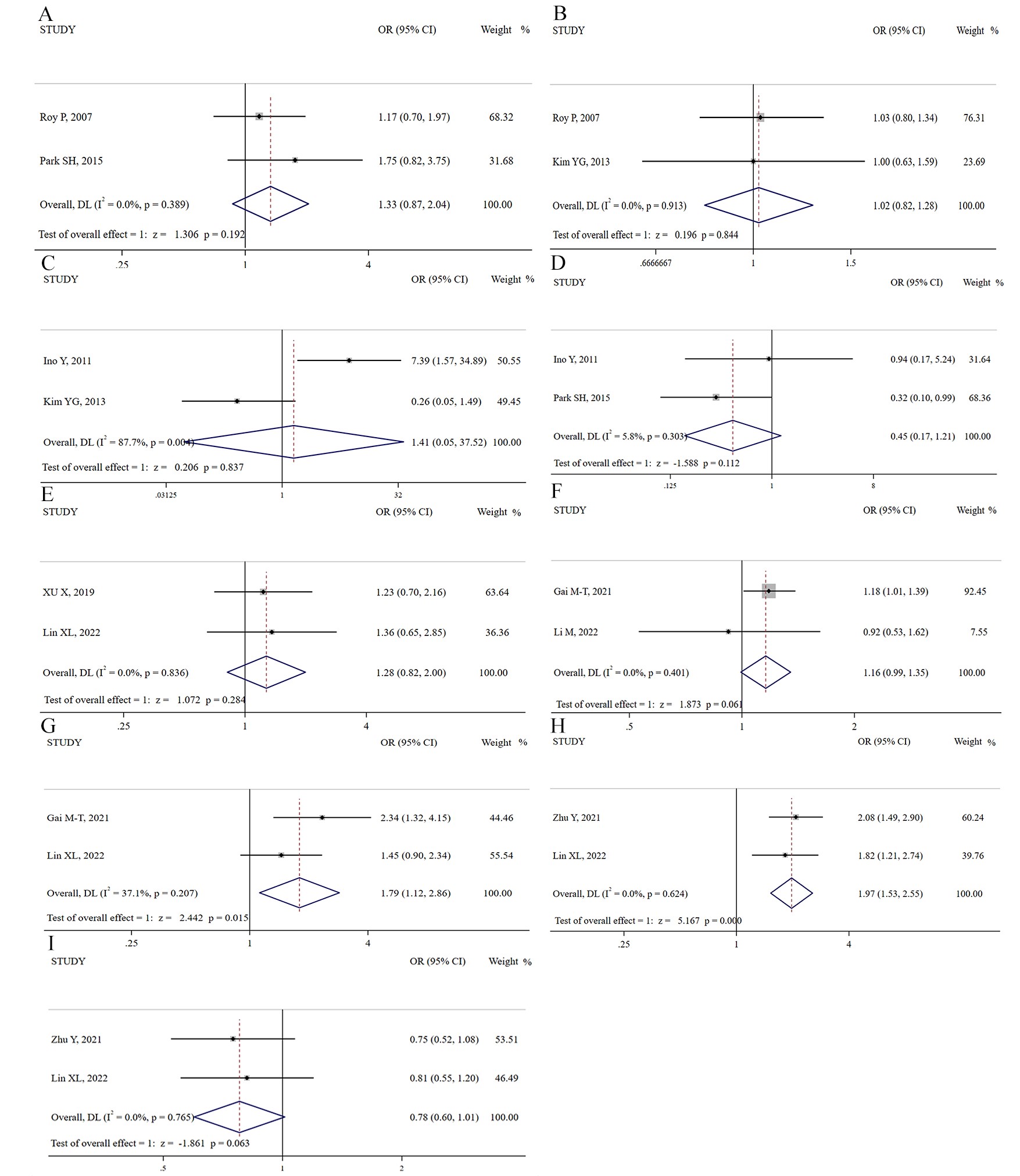

Supplement: Supplementary file 1 [file 2153-8174-25-12-458-s1.zip › Supplementary Fig. 14.jpg]

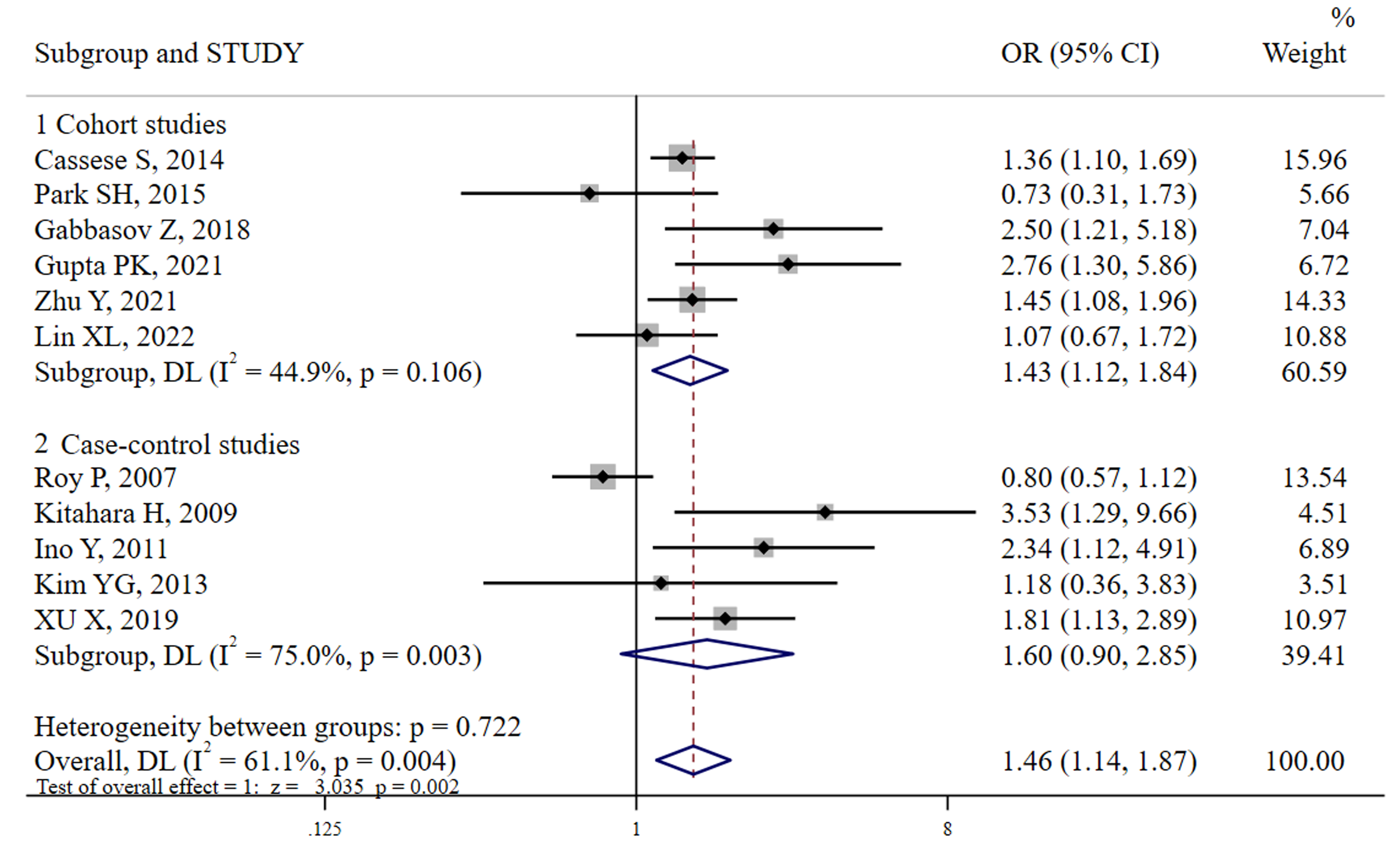

Supplement: Supplementary file 1 [file 2153-8174-25-12-458-s1.zip › Supplementary Fig. 2.jpg]

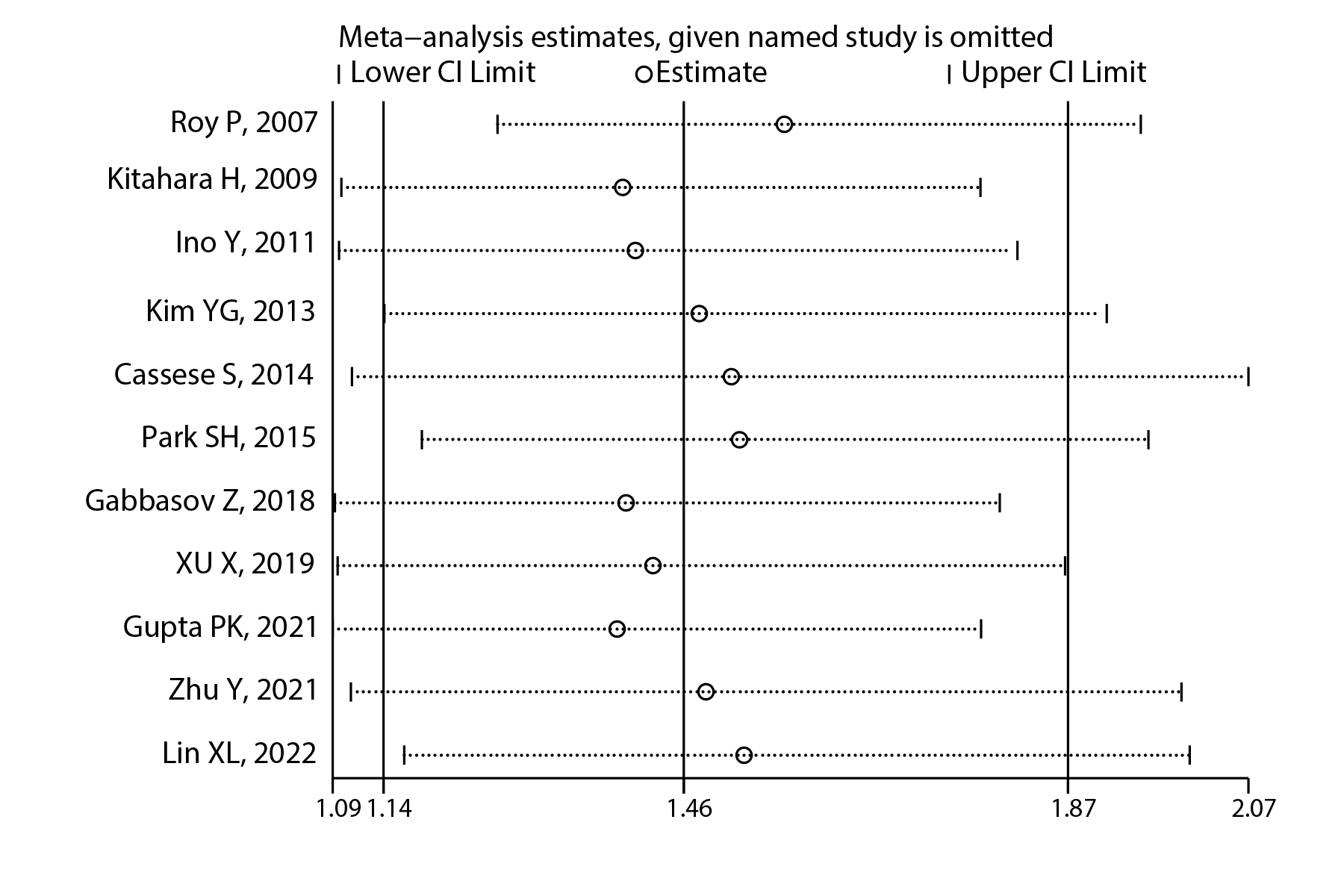

Supplement: Supplementary file 1 [file 2153-8174-25-12-458-s1.zip › Supplementary Fig. 3.jpg]

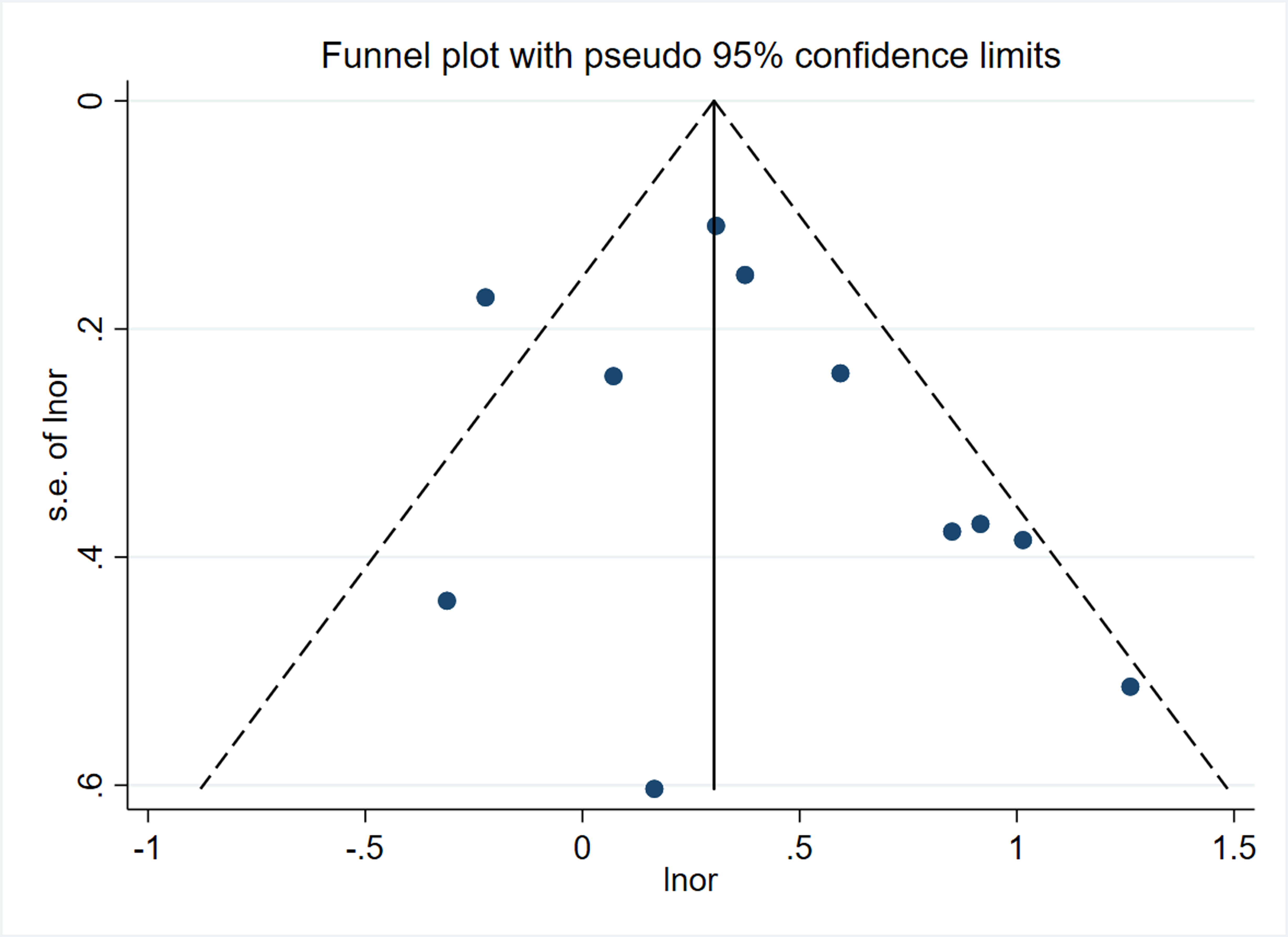

Supplement: Supplementary file 1 [file 2153-8174-25-12-458-s1.zip › Supplementary Fig. 4.jpg]

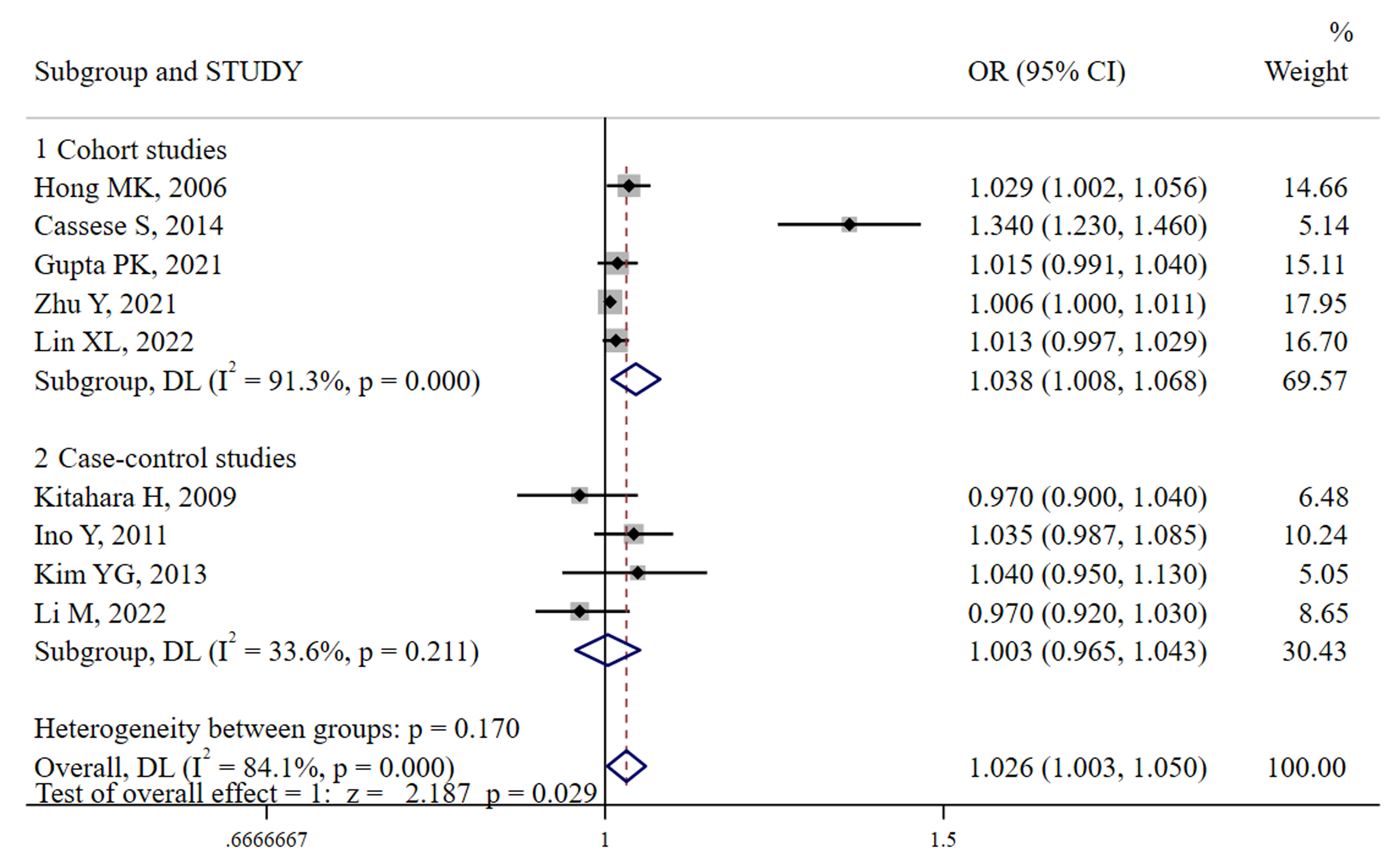

Supplement: Supplementary file 1 [file 2153-8174-25-12-458-s1.zip › Supplementary Fig. 5.jpg]

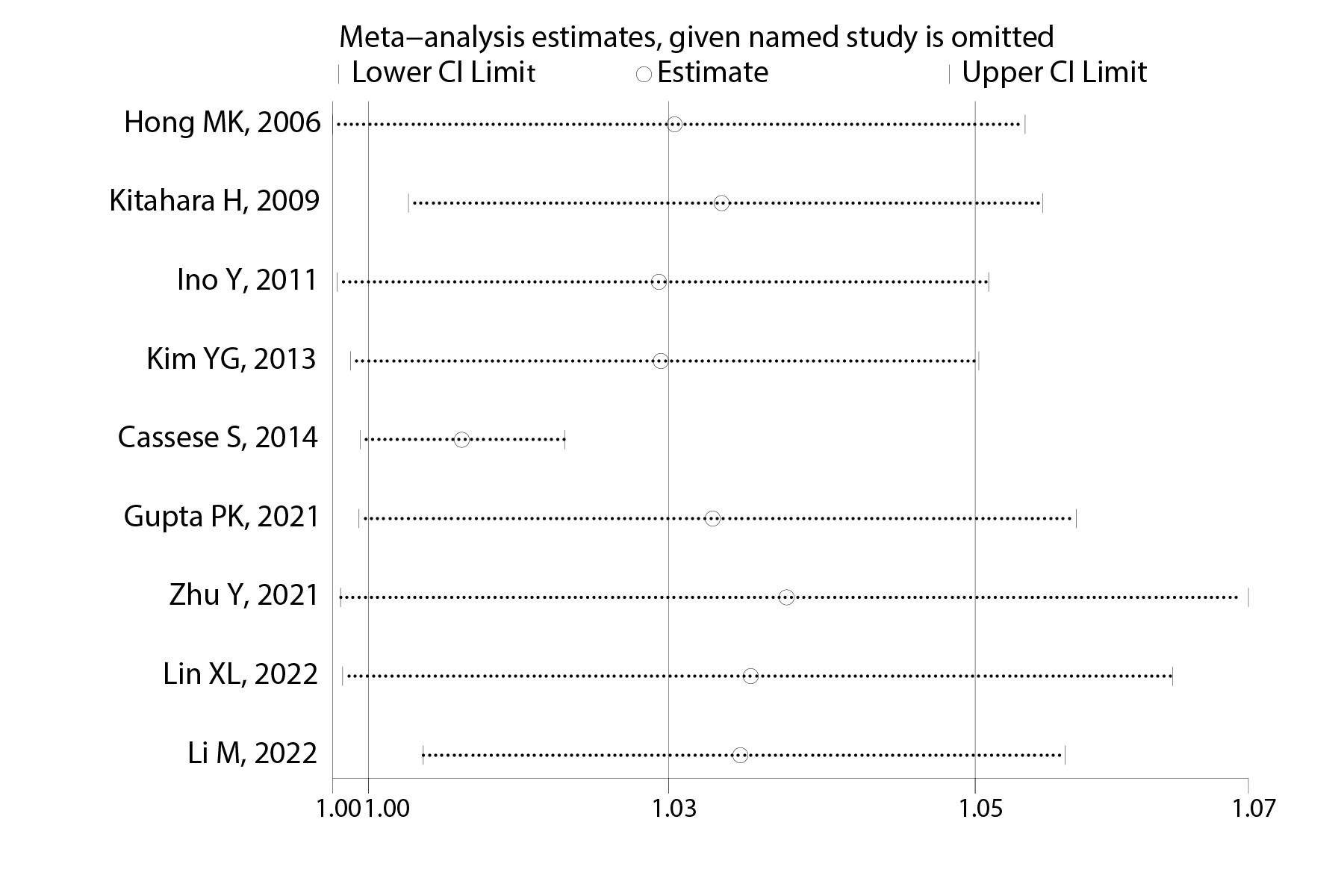

Supplement: Supplementary file 1 [file 2153-8174-25-12-458-s1.zip › Supplementary Fig. 6.jpg]

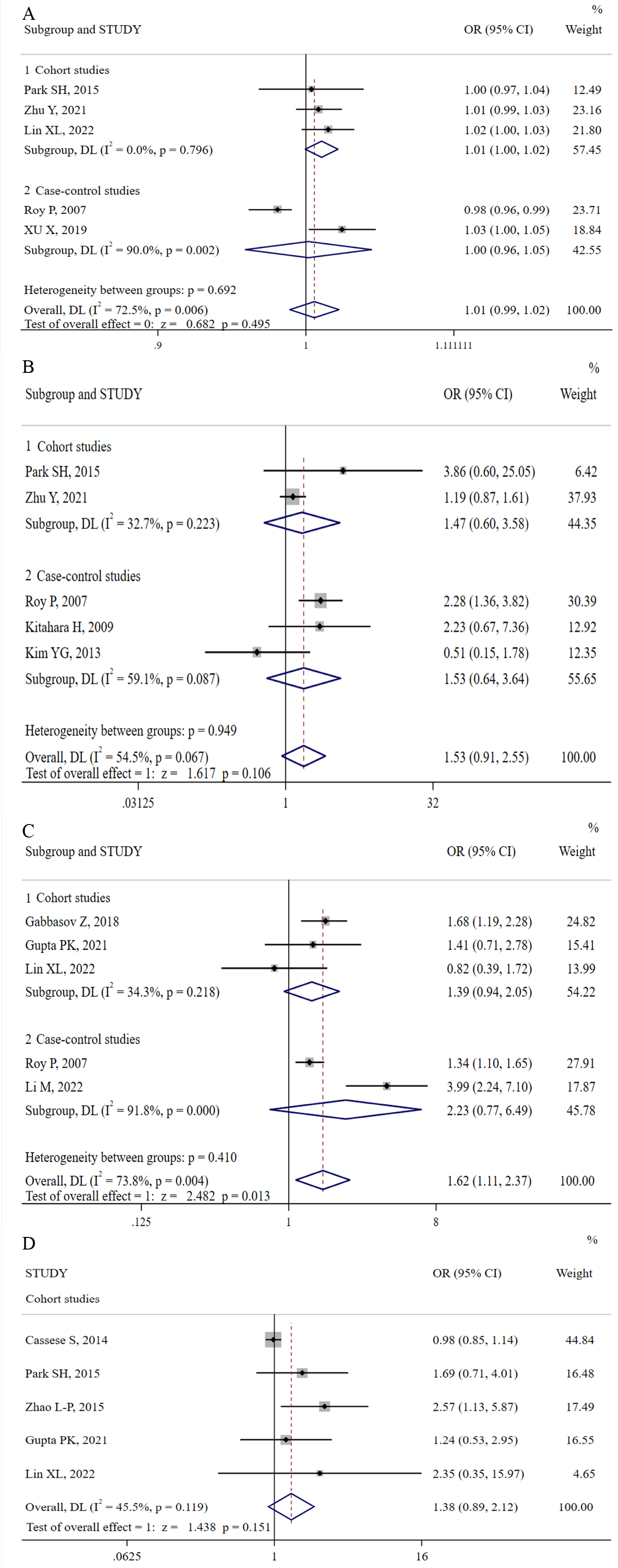

Supplement: Supplementary file 1 [file 2153-8174-25-12-458-s1.zip › Supplementary Fig. 7.jpg]

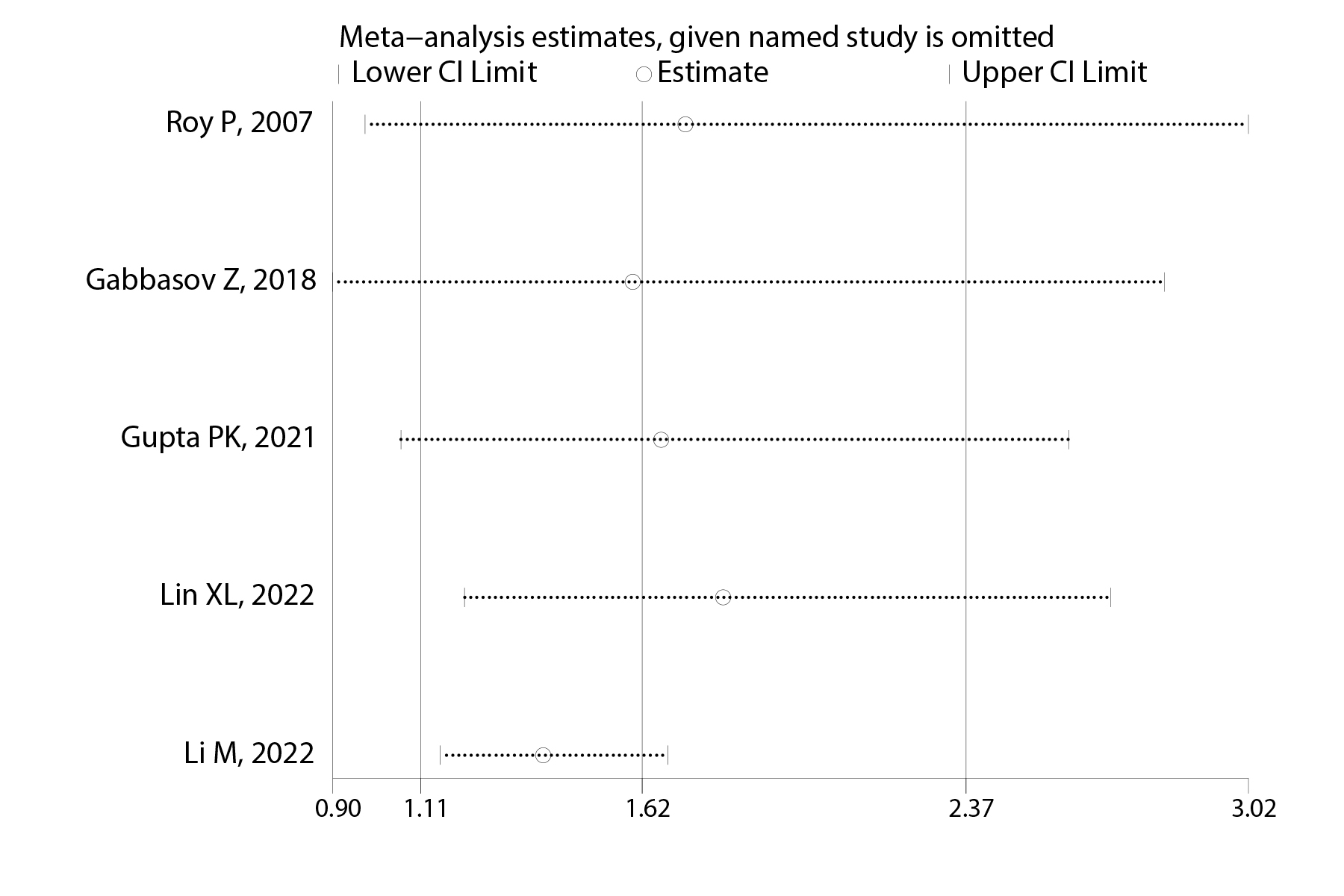

Supplement: Supplementary file 1 [file 2153-8174-25-12-458-s1.zip › Supplementary Fig. 8.jpg]

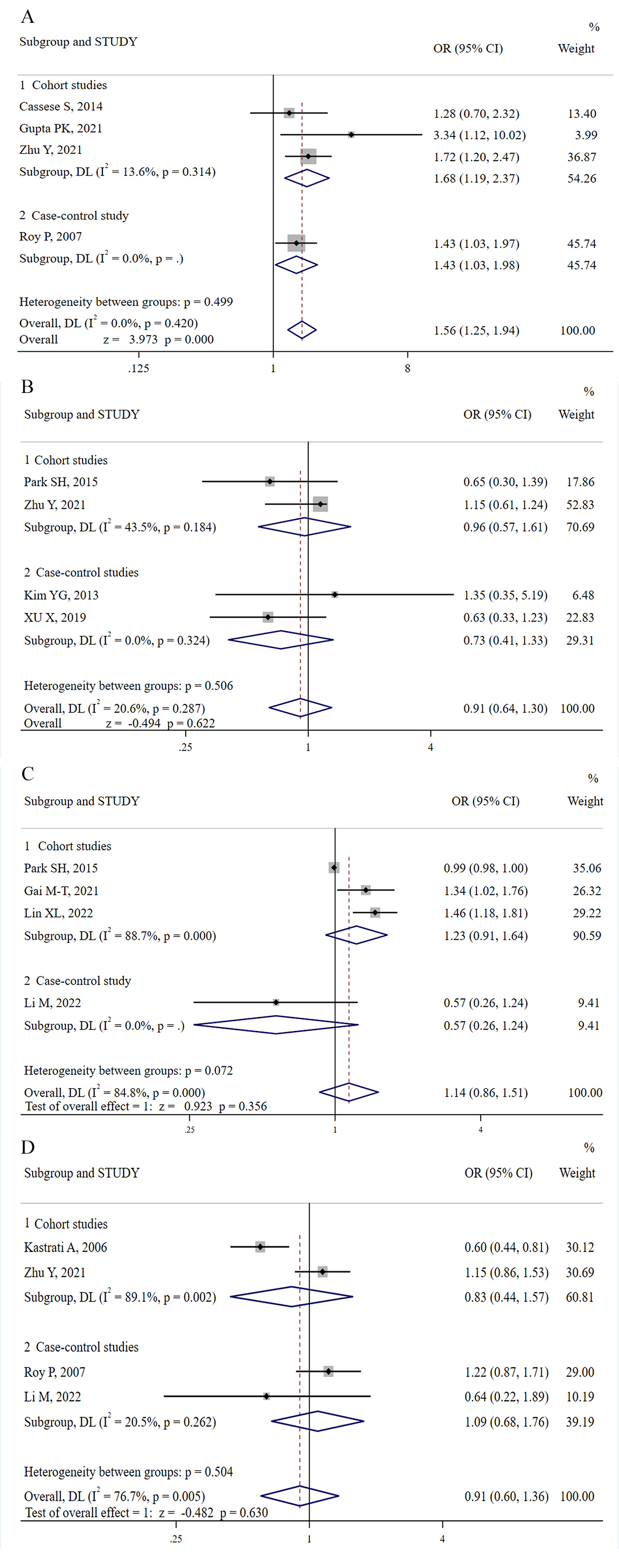

Supplement: Supplementary file 1 [file 2153-8174-25-12-458-s1.zip › Supplementary Fig. 9.jpg]
